# Ecosystem‐Centered Robot Design: Toward Ecoresorbable Sustainability Robots (ESRs)

**DOI:** 10.1002/advs.202509194

**Published:** 2025-12-19

**Authors:** Tülin Yılmaz Nayır, Yuan Fang, Consuelo Contreras, Andrew K. Schulz, Florian Hartmann

**Affiliations:** ^1^ Biomimetic Materials and Machines Group Max Planck Institute for Intelligent Systems 70569 Stuttgart Germany; ^2^ Environmental Engineering Department Faculty of Engineering Gebze Technical University Kocaeli 41400 Turkey; ^3^ Haptic Intelligence Department Max Planck Institute for Intelligent Systems 70569 Stuttgart Germany

**Keywords:** biodegradable polymers, biodegradable robots, ecosystems, soft robotics, sustainability robots

## Abstract

The deployment of robots and sensors across diverse ecosystems supports ecological monitoring, nature conservation, and exploration. However, retrieving these machines is often impractical or economically infeasible, posing risks to ecosystems through pollution, physical damage, and waste generation. To alleviate these risks, the development of transient systems from biodegradable materials represents a promising solution, enabling them to decompose harmlessly after use. Robots made from soft or functional polymers exhibit a unique potential in solving this challenge by drawing from a wide range of biomaterials, while simultaneously benefiting from intrinsic adaptability. Despite significant progress in the development of sustainable soft robotics, the influence of specific ecosystems on biodegradation is frequently overlooked. The environmental context is essential, as biodegradation depends largely on environmental factors unique to each ecosystem. In this review, a comprehensive overview of various ecosystems relevant to robot deployment is provided, offering critical context for assessing sustainability and deriving principles for ecosystem‐centered robot design. Co‐developing materials and sustainability robots with an understanding of their operational ecosystems paves the way for environmentally friendly machines, which are named ecoresorbable sustainability robots (ESRs), that coexist harmoniously with nature.

## Graphical abstract

Robots exploring natural ecosystems can support monitoring and conservation, but must adopt ecosystem‐centered design to avoid pollution, waste, and damage. This review proposes guidelines for co‐designing ecoresorbable sustainability robots (ESRs), uniting materials, robotics, and ecological contexts in a single framework. ESRs are envisioned to operate harmoniously in ecosystems and decompose harmlessly at the end of life.

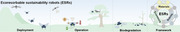

## 1 Introduction

**Figure 1:**
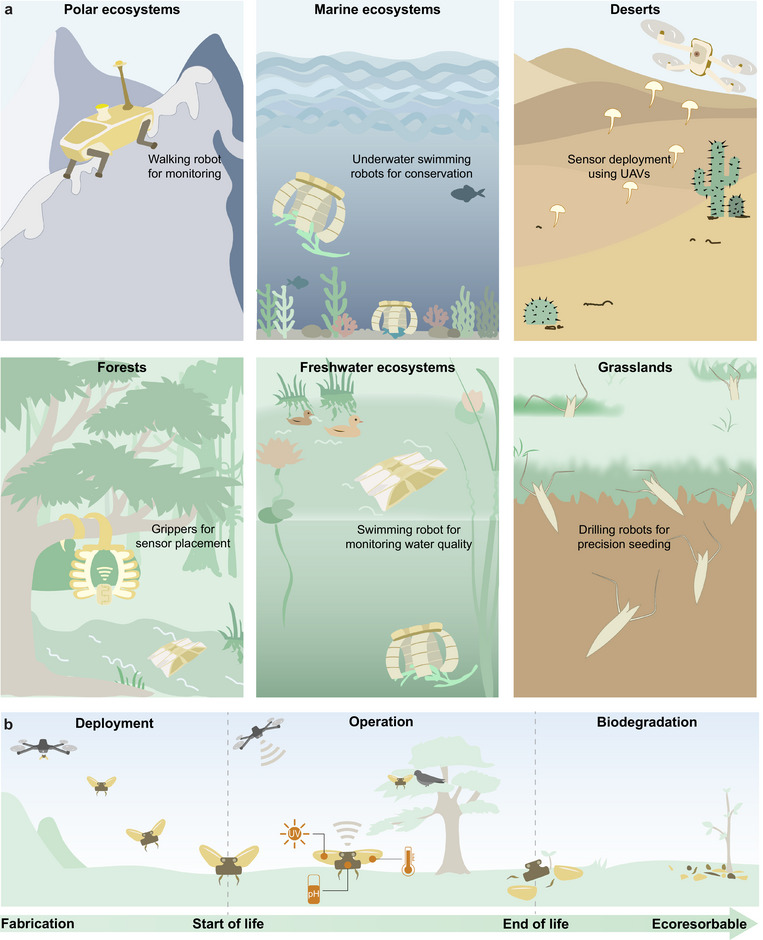
Diverse sustainability robots deployed across a wide range of ecosystems. a) Robots have the potential to be widely applied in ecosystems for assisting with environmental monitoring and nature conservation. For instance, remote‐controlled swimming robots developed by Wang et al. have shown the capabilities of underwater propulsion and manipulation, demonstrating their potential to collect marine trash.^[^ ^28^ ^]^ Also, hygroscopic grippers have been deployed to mount sensors in the canopy of trees for temperature, humidity, and barometric pressure sensing.^[^ ^29^ ^]^ In grasslands, self‐burying robots that carried seeds were dispersed to enhance the seeding success rate.^[^ ^30^ ^]^ The difficulty of retrieving robots in some ecosystems leads to the requirement for biodegradable technologies. b) Considering the diversity of ecosystems, appropriate biodegradable technologies should be developed and deployed following an ecosystem‐centered design approach. Robots that are deployed to a specific ecosystem should operate without causing harm to the flora and fauna, and have the ability to be ecoresorbable after they are no longer in use.

Distributing instruments to the environment has emerged as an effective tool in the fight for maintaining our planet's health. Human's far‐reaching impact on the world's ecosystems resulted in their degradation and in a decline of biodiversity, a concept known as anthropogenic change, which is mainly caused by invasive species, pollution, improper waste treatment, and over‐exploitation of resources. Environmental analyzers and machines can help to improve air, water, and soil quality and to effectively manage natural resources (**Figure** **1**a).^[^ ^1^, ^2^ ^]^

However, the deployment of these technologies must be guided by a commitment to sustainability itself. At the end of use, the retrieval and proper disposal of technology is often impractical or economically unfeasible in certain ecosystems.^[^ ^3^ ^]^ This problem extends to human‐centric environments, where retrieval and recycling would be a sustainable solution, but improper treatment often results in waste ending up in landfills or natural ecosystems.^[^ ^4^ ^]^

To address this dilemma, researchers have turned to the development of transient technologies designed to vanish without a trace.^[^ ^5^, ^6^, ^7^, ^8^ ^]^ From a sustainability perspective, it is crucial that these technologies degrade in an environmentally benign manner, decomposing into nontoxic compounds that do not harm ecosystems. As a result, polymers–both synthetic and biosourced–have emerged as a key material class for transient systems. The cornucopia of available biopolymers has great potential for biodegradable technology, leveraging their abundance, diverse material properties, and tunability to create sustainable solutions.^[^ ^9^, ^10^ ^]^ Additionally, the emergence of electronic and robotic technologies based on conformable and soft materials^[^ ^11^, ^12^ ^]^ has driven a paradigm shift in machine design, moving away from traditional metal‐based architectures, and further accelerated the advancement of transient devices and machines. It has sparked the development of sustainable soft robotics,^[^ ^13^, ^14^ ^]^ edible robotics^[^ ^15^ ^]^ and electronics,^[^ ^16^ ^]^ and bioresorbable electronics for in‐vivo use.^[^ ^17^, ^18^, ^19^, ^20^ ^]^

However, the biodegradability of these technologies is highly dependent on the environmental conditions in which they are deployed to. Temperature, chemical composition, availability of oxygen, and microorganisms for enzymatic degradation all affect the rate and extent of biodegradation.^[^ ^21^, ^22^, ^23^, ^24^ ^]^ For example, conditions and microorganisms to degrade polylactic acid (PLA) are not found throughout all environments. Even though PLA is called biodegradable, it needs temperatures close to its glass transition temperature and specific enzymes to degrade. While it efficiently decomposes within the human body or during industrial composting (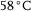 and in soil), it does barely degrade in aquatic environments where temperatures are colder, the microorganisms are different, and there is less oxygen available for the microorganisms to metabolize the material.^[^ ^25^, ^26^, ^27^ ^]^

In this review, we discuss biodegradability in the context of environmental conditions across various ecosystems and how robots can be deployed to these ecosystems (Figure 1b). First, we introduce the three core concepts of ecosystem‐centered design for ecoresorbable sustainability robots (ESRs): 1) the diversity of ecosystems, 2) a background on biodegradability and ecoresorbability, and 3) robotics deployment to ecosystems. We proceed to assess ecosystems around the globe and give them scores on their ease of access and their efficacy to support biodegradability, guiding material and robot design parameters. This allows categorization of ecosystems in four quadrants that enable researchers to derive strategies for the end‐of‐life treatment of robots in diverse ecosystems. For researchers, in particular for those coming from engineering disciplines, we provide a starting point for the design of ESRs by providing a proposed framework that incorporates a biodegradable material loop, robotic design loop, and an ecoresorbable robotic loop. We propose this framework along with a case study of an ESR deployment to a warm desert ecosystem. We then highlight examples of biodegradable functional materials and soft robots with the potential to be deployed in nature. Finally, we explore the challenges and opportunities presented by the development of sustainable robotic technologies purpose‐built for use in natural ecosystems that will lead to the realization of ESRs. By taking a comprehensive approach to biodegradability in the context of environmental conditions, we aim to provide a valuable resource for researchers working in the field of biodegradable technologies and sustainability robots.

## 2 Classification of Ecosystems

Every ecosystem presents unique challenges and advantages for biodegradation, making it essential to classify them based on how environmental conditions facilitate biodegradation. By reviewing these ecosystems and summarizing their environmental features, we can gain a deeper understanding of the design and material choices that must be considered for truly ecoresorbable robotic systems. We take a snapshot of each ecosystem, typically using data from the period 2000 to 2015 and, in some cases, more recent data, to allow for comparisons and contrasting of these specific systems, noting that conditions fluctuate because of climate change. In general, we differentiate between natural ecosystems, including terrestrial and aquatic, as well as artificial ecosystems, which are all subdivided into characteristic ecosystems, which are discussed more in depth in the Supporting Information. We include a brief summary of most important features for each ecosystem class.

### Terrestrial Ecosystems

This class encompasses 12 natural terrestrial ecosystems, 11 of which are described and grouped by Cheng et al.,^[^ ^31^ ^]^ including polar ecosystems, four types of grasslands, three types of deserts, three types of forests, and additionally caves. Polar ecosystems refer to the latitudinal regions where the mean annual biotemperature is between 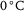 and 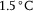, such as the High Arctic and Antarctica.^[^ ^32^ ^]^ Similar conditions also occur in high‐altitude zones, such as the nival belt above 4750 m and the high Tibetan plateau.^[^ ^33^ ^]^ In contrast to these cold ecosystems, grasslands are characterized by grass‐dominated vegetation, including grasses, sedges, and herbaceous plants as the primary types. These ecosystems include tundras and alpine steppes, steppes, temperate humid grasslands, and savannahs. They experience significant seasonal variations, such as cold winters and warm summers, or alternating wet and dry cycles. Deserts, which are polyextreme environments, are characterized by large temperature fluctuations (between 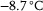 and 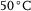), extremely low annual precipitation, and scarce vegetation. Depending on thermal characteristics, they can be broadly classified as warm, cold, or semi‐deserts. In contrast, forests have a unique structure of vegetation with vertically stratified canopies comprising emergent, canopy, understory, and ground layers. Humid microclimates are found inside these forests and are characterized by lower annual average wind speeds, higher humidity, and moderate temperature fluctuations. Depending on the climatic conditions in terms of temperature and precipitation, forests are classified as tropical, temperate, or subtropical. Caves, the last terrestrial ecosystem type, are characterized by total darkness or low‐levels of light, high humidity, and sharply reduced levels of oxygen, from the entrance to deeper, narrower passages.^[^ ^34^ ^]^ These ecosystems are located across various climatic zones, from tropical to cold continental climates.^[^ ^35^ ^]^

### Aquatic Ecosystems

Water occupies approximately 71 % of the Earth's surface in various forms, including oceans, seas, rivers, lakes, and underground water networks.^[^ ^36^ ^]^ Here, we categorize bodies of water by their salinity (saline or fresh water) and depth (water surface or underwater). Saline waters, with a salinity typically ranging from 34 to 36 parts per thousand (ppt), account for approximately 97.5 % of the world's water in the form of seas and oceans.^[^ ^36^ ^]^ Surface and deep sea environments, both of which are part of saline waters, have different physical conditions and nutrient sources. Surface water receives more sunlight and oxygen than underwater regions, resulting in differences in temperature and oxygen between the surface and the deep sea. While 5% to 10 % of solar radiation is reflected at the water's surface, light reaches through the top layers of water. Due to the absorption spectrum of water, UV light in the range of 320 nm to 400 nm exhibits the highest penetration, where the radiance attenuates to 10 % of its original intensity at a depth of 38 m.^[^ ^37^ ^]^ Moreover, sunlight on the surface enables photosynthesis, which generates organic matter and supports biologic activity. In contrast, the dark, oxygen‐poor deep sea depends on sinking inorganic particles, dead cells, and other detritus from the upper layers.^[^ ^38^ ^]^ The deep sea provides a habitat for coral reefs, which are large underwater structures formed by living organisms and characterized by high levels of biodiversity.^[^ ^39^ ^]^ Fresh water is primarily stored in polar and groundwater ecosystems. Only about 0.25 % of fresh water is held in rivers and lakes.^[^ ^36^ ^]^ Although small in quantity, these ecosystems are essential for the survival of humans, flora, and fauna.

### Artificial Ecosystems

Artificial ecosystems are natural ecosystems that have been augmented by humans to fulfill human‐centric social, economic, or environmental needs. Unlike natural ecosystems, which change through ecological succession ^[^ ^40^ ^]^ (including both ecological and anthropogenic factors), artificial ecosystems built for specific purposes to fulfill human‐centered needs, such as food production, urban development, and climate resilience. These ecosystems have some or all of their environmental factors augmented and controlled to provide benefits for their intended purpose. We chose specific sites for the artificial ecosystems because they can be created in almost all of the aforementioned natural ecosystems (e.g., aquaculture, cactus farming, and grassland farming). We selected a Nigerian farmland as an example of agricultural fields, and an open German lignite mine as an example of mines. This review does not directly cover urban ecosystems, as they are one of our reference points for assessing the accessibility of ecosystems. Additionally, end‐of‐life scenarios are typically outsourced from urban to other (artificial) ecosystems, such as landfills or waste treatment facilities.^[^ ^41^ ^]^

To systematically classify ecosystems for the sustainable deployment of robots, we need to assess the conditions that influence their ability to support biodegradation, as well as classify their accessibility. In the following sections, we discuss biodegradability in relation to the ecosystems where robotic deployment is possible. We provide a detailed examination of the typical conditions within these ecosystems and discuss how they either support or hinder biodegradation. This classification will offer a strategy for designing sustainability robots that are optimized for either recovery or biodegradability. We proceed to introduce biodegradability and ecoresorbability in the context of these diverse ecosystems, along with the environmental factors, mechanisms, and standard methods involved in the biodegradation of materials.

## 3 Biodegradability and Ecoresorbability in the Context of Ecosystems

### 3.1 Terms that Describe Biodegradability

In literature, the term biodegradable and related terms, such as compostable, edible, bioresorbable, and ecoresorbable, are often used interchangeably despite having distinct meanings. The main difference between these terms lies in the environment and its conditions in which the degradation process occurs. In this section, we explain the context in which these terms are used to provide clarity for inexperienced readers.

In general, the term transient is used to describe systems that purposefully disappear after use through dissolution, disintegration, degradation, or digestion.^[^ ^5^ ^]^ This term does not specify a particular degradation mechanism or environment. However, in many studies, transient systems are defined as materials that disintegrate into non‐toxic compounds.

Materials that are converted into non‐toxic compounds by enzymatic processes, facilitated by microorganisms, can be described as biodegradable.^[^ ^42^ ^]^ The term is more specific than *transient* because it provides information about the degradation process and outcome. However, it does not specify the specific environments and conditions in which the biodegradation occurs.

Materials that are decomposed by microorganisms under controlled heating and aeration are termed compostable. The conditions and environment (compost) are standardized in industrial composting processes, which accelerate material decomposition.

In the context of the human body, the terms edible and bioresorbable are commonly used. ***Edible*** materials can be safely consumed and naturally digested by humans. This term is also sometimes used to describe biocompatible compounds, such as gold thin films, that can pass through the digestive system without harm due to their small size.^[^ ^43^ ^]^ ***Bioresorbable*** materials can vanish within the human body.^[^ ^42^ ^]^ The term is primarily used in a medical context to describe implantable materials and technology that can be fully absorbed by the body after insertion.

Materials that are compostable, edible, or bioresorbable may have similar chemical structures. However, the conditions necessary for their decomposition are distinct in each case. For example, a compostable material may not be bioresorbable, and a bioresorbable material may not be edible. Additionally, not all biodegradable materials necessarily decompose in natural ecosystems within practical time scales (e.g., materials such as wood can take decades). For materials that can decompose in an ecosystem without harming it, the term ecoresorbable is used. Ecoresorbable materials enable the development of functional devices that operate for a defined period and then disappear within their application ecosystem. Transient sensing devices for environmental monitoring, for example, can be fabricated from conductors (e.g., Mg, Zn, or activated carbon), semiconductors (e.g., Si, ZnO, or MgO) and insulating encapsulants (e.g., Si, cellulose, or polysaccharides). These materials are capable of complete dissolution after a defined period, leaving no harmful residues.^[^ ^44^ ^]^

### 3.2 Biodegradation Mechanisms

**Figure 2:**
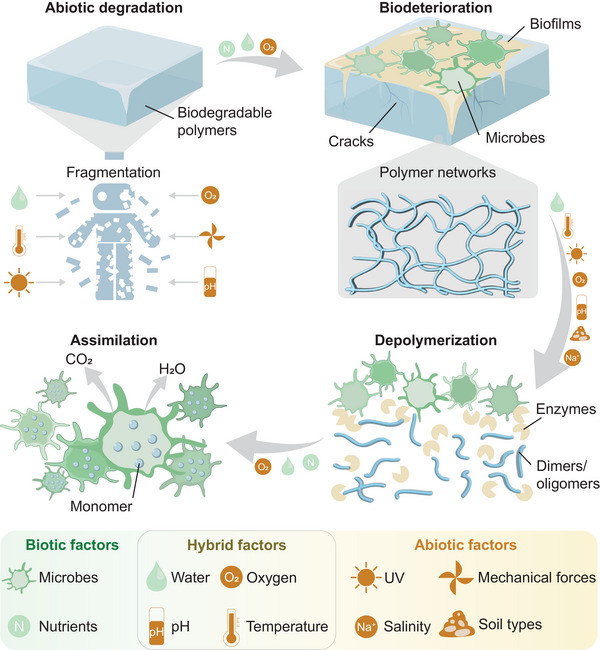
Mechanisms and major factors influencing biodegradation. The biodegradation process includes abiotic and biotic factors. Initially, materials are fragmented into smaller pieces with the assistance of abiotic factors. Subsequently, a biofilm is secreted by microorganisms on the material's surface, initiating biodeterioration, followed by the depolymerization of macromolecules into monomers, which are eventually assimilated by the microorganisms.

Biodegradation occurs through both biological (biotic) and non‐biological (abiotic) processes, summarized in **Figure** **2**. While microorganisms primarily induce biodegradation, the contribution of abiotic factors to this process should not be neglected. Biotic degradation involves the enzymatic actions of microorganisms and leads to the production of CO_2_, CH_4_, and H_2_O under aerobic and anaerobic conditions. Abiotic degradation can occur through mechanical, light, thermal, and chemical degradation (hydrolysis) processes, resulting in the production of smaller fragments with lower molecular weights. This fragmentation process facilitates faster biodegradation, enabling further breakdown by microorganisms. A comprehensive discussion of biodegradation mechanisms can be found in several reviews.^[^ ^21^, ^22^, ^23^, ^24^ ^]^ In summary, the biodegradation process consists of three stages:

1. *Biodeterioration*: Biodegradation begins with the formation of a biofilm on the material's surface. Microorganisms secrete substances that enable them to adhere to the material surface and penetrate the porous structure of the material, thereby weakening its durability and damaging its mechanical properties.
2. *Depolymerization*: Macromolecules with high molecular weights break down into smaller molecules through the cleavage of chemical bonds. This process allows the molecules to cross the microorganisms' cell walls, which would otherwise be impermeable to molecules with high molecular weights.
3. *Assimilation*: The monomers produced in the depolymerization step are integrated into microbial cells, enabling the microorganisms to grow and reproduce.

Whether a material is ecoresorbable depends not only on its intrinsic properties, but also on the environmental conditions present in the ecosystem. This section discusses the environmental factors that influence biodegradation mechanisms.

### 3.3 Environmental Factors

Biotic degradation is primarily influenced by nutrient availability and microbial abundance, which are both key biotic factors. Abiotic degradation is driven by physical factors (e.g., UV exposure and mechanical forces), as well as chemical factors (e.g., soil type, and salinity). Additionally, temperature, oxygen, and pH affect both biotic and abiotic degradation processes. These are referred to as hybrid factors because they enable microbial life and promote hydrolysis.

*Concentration and availability of nutrients*: The availability of nutrients such as carbon (C), nitrogen (N), and phosphorus (P) are essential for the survival and activity of microorganisms, making their availability a growth‐limiting factor. These elements, as well as minerals, are crucial for various cellular processes, including energy production, protein synthesis, and nucleic acid synthesis, which are necessary for new cell production. In ecosystems, polymers are not typically the main source of nutrients, but the presence of other nutrient sources (e.g., organic matter) can enhance microbial digestion and support the biodegradation process.^[^ ^45^ ^]^

*Abundance of microorganisms*: Biodegradation requires microorganisms, which influence the rate of biodegradation through their concentration and population diversity. A high microbial concentration accelerates biodegradation by increasing enzyme production, which is responsible for biofilm formation. Furthermore, microbial diversity is essential for degrading different types of materials, as various species of microbes are capable of degrading distinct polymers. In natural environments, bacteria and fungi are among the most widely distributed groups of microorganisms involved in polymer degradation.^[^ ^46^ ^]^

*Water availability*: Water is an essential parameter for microbial growth and reproduction as it provides a suitable environment for the diffusion of nutrients, enzymes, and metabolic products.^[^ ^22^ ^]^ Additionally, water plays a crucial role in the hydrolysis process, facilitating the cleavage of bonds and reducing the molecular weight of polymers, thereby enhancing their accessibility to microorganisms.^[^ ^47^ ^]^

*Temperature*: Temperature influences biodegradation through inducing thermal degradation and accelerating hydrolysis, with distinct temperature regimes changing biodegradation mechanisms.^[^ ^48^ ^]^ Below the polymer's glass transition temperature (*T*_g_), physical aging occurs, leading to increased stiffness and brittleness, as well as reduced permeability of fluids^[^ ^49^ ^]^ or gases.^[^ ^50^ ^]^ Between *T*_g_ and the melting temperature (*T*_m_), polymers undergo dimensional changes and decomposition of chains with low molecular weight. At temperatures higher than *T*_m_, melting and loss of structure occur.^[^ ^51^ ^]^ Furthermore, elevated temperatures enhance hydrolysis by increasing polymer chain mobility and the reactivity of hydrolysable bonds. This increased reactivity accelerates chain scission and reduces molecular weight. However, temperatures above 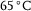 are generally unfavorable for microbial activity, as such conditions lead to a decrease or interruption of microbial activity.^[^ ^52^ ^]^

*Ultraviolet (UV) exposure*: Polymers are susceptible to photo‐oxidative degradation, a process induced by solar radiation.^[^ ^22^ ^]^ When UV light is absorbed, free radicals form in the polymer, which leads to the degradation of the polymer's mechanical properties through chain scission and a reduction in molecular weight (*M*_*w*_). The most effective wavelength range for photodegradation is between 250 nm to 340 nm.^[^ ^53^ ^]^

*Oxygen availability*: Environments can be classified based on oxygen availability as aerobic (with free oxygen present), anaerobic (without free oxygen), or anoxic (without free oxygen but with oxygen‐containing compounds such as NO_3_), which significantly influences the rate of biodegradation. Aerobic bacteria degrade plastics more rapidly in oxygen‐rich environments, whereas anaerobic bacteria operate more slowly in oxygen‐limited environments, such as landfills or deep‐sea sediments.^[^ ^46^ ^]^ In addition to biotic degradation processes, oxygen and its forms (O_2_ and O_3_) can initiate chemical degradation through autoxidation, forming highly reactive free radicals that break polymer chains. This process results in significant changes of the material's structure and properties.^[^ ^54^ ^]^

*Mechanical forces*: Mechanical forces, including compression and tension, induce fatigue in materials, ultimately leading to fragmentation.^[^ ^21^ ^]^ Fragmentation accelerates the biodegradation rate by increasing the material's surface area, thereby making it more accessible to microorganisms. However, if environmental conditions inhibit microbial activity, the fragmentation of polymers results in the formation of microplastics that pollute the ecosystem. Wind is an example of such a force, causing the movement and friction of objects and slowly leading to fragmentation. Another force that facilitates this process, and does so more rapidly, is fire. The heat generated by fire effectively melts or incinerates materials, thereby enhancing their susceptibility to biodegradation by microorganisms.

*pH values*: The pH level of an environment impacts both biotic and abiotic degradation processes. Neutral conditions are generally favorable for microbial activity and support biotic degradation. However, certain microorganisms, such as extremophiles, have adapted to acidic or alkaline environments. Abiotic degradation is significantly accelerated in acidic and alkaline environments due to increased hydrolysis. Furthermore, extreme pH conditions can result in distinct degradation mechanisms in materials. For example, the degradation of polycaprolactone (PCL) films is caused by surface erosion in alkaline (pH 13) media and bulk erosion in acidic (pH 1) media.^[^ ^55^ ^]^

*Soil properties*: Soil is formed from a mixture of particles—characterized into clay (diameter < 2μm), silt (diameter from 2μm to 50μm), and sand (diameter from 0.05 mm to 2 mm)—as well as organic matter and water. These components collectively influence soil texture. The size distribution of matter aggregates in soil is influenced by agricultural practices such as cultivation, drainage, fertilization, and compaction. These practices affect water, gas, and heat transfer in the soil. For example, sandy soils tend to facilitate better gas diffusion, whereas clay soils are often poorly aerated.^[^ ^45^ ^]^ However, clay soils provide a favorable environment for microbial activity due to the abundance of essential cations, such as ${\mathrm{NH}}_{4}^{+}$, K^+^, and Mg^2 +^.^[^ ^22^ ^]^

*Salinity*: In saline waters, the osmotic effect reduces water diffusion into the polymer, resulting in decreased water availability. The reduced water availability is slowing down the biodegradation process.^[^ ^56^ ^]^

Environmental factors determine the rate at which materials biodegrade. To assess a material's biodegradability, it can be tested in standardized tests that simulate specific factors and conditions, or biodegradability indicators are examined in real environments. In the following section, we provide an overview of test methods and biodegradability indicators.

### 3.4 Assessment and Indicator for Biodegradability in Materials

The environmental conditions vary significantly from one ecosystem to another, as they were defined in Section 2. Therefore, biodegradability should be tested under the specific conditions of the ecosystem where a robot is going to be deployed. Standardized methods (e.g., ISO, EN, and ASTM) are defined to address this difficulty by covering various disposal scenarios and controlling conditions such as medium, temperature, and duration of the test. This strategy enables comparison between materials. Testing for indicators of biodegradation, such as weight loss, allows materials to be analyzed in field tests or under conditions that are difficult to simulate in a standardized test. Several studies have reviewed standardized methods and indicators for testing for biodegradability in various environments and for simulated and real conditions. For example, Folino et al. provided a comprehensive review of a wide range of standards for terrestrial (soil and compost) and aquatic environments,^[^ ^57^ ^]^ while Lavagnolo et al. focused exclusively on standards for aquatic environments.^[^ ^58^ ^]^ In this section, we provide some examples.

#### Standardized Test Methods

Most standards for measuring biodegradability were introduced to evaluate waste disposal methods. If it is feasible to retrieve a robot from its deployment site, it should be disposed of using a suitable waste treatment method. However, this approach requires knowledge of, or control over, waste streams, particularly information on where the technology ends up after its end‐of‐use. Here, we highlight standards for conditions found in solid waste treatment processes, including composting, land filling, and anaerobic digestion, as well as for soil and aquatic environments (**Table** **1**). While these scenarios do not encompass all conditions in ecosystems, these standards provide practical reference points.

**Table 1:** Standards for testing biodegradability. Summary of standardized test methods for assessing biodegradability in various environments. Citations reference either the direct standard or publications where the standard was applied.

Environment	Standardized test method	Method description
Solid waste treatment	ISO 14855‐1:2012 [59], ASTM D5338‐15 [60]	These standards determine the aerobic biodegradability of plastics under controlled composting conditions. The test material is incubated at 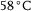 for up to 6 months, and its carbon conversion into CO_2_ is measured.
	ISO 20200 [61]	This standard determines the degree of disintegration of plastics under simulated composting conditions by measuring the mass reduction of test samples.
	ASTM D5526 [62]	This method assesses the anaerobic biodegradation under simulated landfill conditions ( 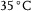 , pH 7.5–8.5), by measuring biogas evolution (CO_2_ and CH_4_).
	ASTM D5511 [63], ISO 15985 [64]	These methods determine the degree and rate of anaerobic biodegradation of plastic materials under low or high soil content in the anaerobic digestion reactor by calculating the volume of produced biogas as a percentage of biodegradation.
Soil	ASTM D5988‐18 [65], ISO 17556:2019 [66]	These methods assess the biodegradation of bioplastic samples buried in prepared soil at 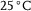 with optimal moisture and oxygen conditions by measuring CO_2_ evolution over time.
	NF U52‐001, UNI 11462, and EN 17033 [57]	These standards assess the impact of plastic degradation in soil. However, unlike others, they also consider the ecotoxicity concept as well as biodegradation.
Aquatic environments	ISO 23977‐1:2020 [67]	This method is designed to evaluate biodegradability of plastic materials exposed to seawater. It quantifies biodegradation degree based on produced CO_2_.
	ISO 23977‐2:2020 [68]	This method is also designed to evaluate biodegradability in seawater, but determines it by measuring the oxygen consumption in a closed respirometer.
	OECD 306 [69]	This standard describes two methods for biodegradability in seawater: one determines the degradation by measuring DOC concentration over the test duration, while the other monitors oxygen consumption.
	ASTM D7991‐15 [70]	This standard evaluates the biodegradability of plastic materials under controlled conditions when exposed to sandy marine sediment.
	OECD 301 [71]	This standard assesses the ready biodegradability of a test material in an aerobic aqueous medium other than saline environment over a 28‐day test period by measuring DOC concentration, or CO_2_ production, or oxygen consumption.
	ISO 14853:2016 [72]	This standard assesses anaerobic biodegradability of plastics treated in anaerobic facilities. The test material is exposed to anaerobic sludge for up to 90 days and biogas (CO_2_ and CH_4_) production is measured.
	ISO 22766:2020 [73]	This standard provides a method for testing plastics in real marine environments; it only describes the physical disintegration, not biodegradation.

Standardized methods are useful for comparing materials, but they are based on simulated terrestrial and aquatic environments or conditions that often do not reflect the actual conditions of the desired ecosystem. While ISO 22766:2020 provides a method for testing plastics in real marine environments, it only describes physical disintegration, not biodegradation. Furthermore, testing new materials for biodegradation can be time‐consuming, particularly when it is unclear whether a material can degrade under certain conditions. In such cases, it may be more feasible to investigate indicators of transient behavior and biodegradation.

#### Indicators of Transient Behavior and Biodegradability

Indicators of transient behavior help to analyze the physical or molecular changes of a material. They can be tested under various conditions and in different environments. However, many indicators, particularly physical ones, may only show transient behavior and do not necessarily indicate whether materials are undergoing biodegradation.

Physical indicators include changes in appearance (e.g., color and opacity), morphology, mechanical properties, and weight loss. Analytical testing methods for these indicators have been defined and applied in the literature,^[^ ^74^, ^75^, ^76^ ^]^ but we briefly summarize the primary techniques and give each technique's abbreviation. Most frequently, the weight reduction of a material is measured to monitor disintegration or fragmentation. In this test, samples are extracted from the test environment at regular time intervals, cleaned, dried, and weighed to determine weight loss. Field emission scanning electron microscopy (FE‐SEM) is used to visualize changes in the material's surface morphology and determine if surface erosion is occurring, such as through hydrolysis. Tensile or compression tests evaluate changes in molecular structure that affect macroscopic properties such as Young's modulus. Similarly, thermogravimetric analysis (TGA) can be used to determine the material's thermal stability and assess its behavior from low to high temperatures. To understand the micromolecular changes in a material, the change in molecular weight of polymers can be measured using gel permeation chromatography (GPC). The chemical composition can be evaluated by Fourier transform infrared spectroscopy (FTIR), and the crystal structure of materials can be determined by X‐ray diffraction (XRD) analysis.

Indicators of microbial activity are useful for determining whether microorganisms are involved in the degradation process. In aquatic environments, for example, indicators such as biochemical oxygen demand (BOD), CO_2_ evaluation, biogas production (CO_2_ and CH_4_), and clear zone formation analysis are commonly used to assess microbial activity during biodegradation.^[^ ^57^, ^58^ ^]^ In soil environments, weight loss and identification of microorganism through ribosomal ribonucleic acid (rRNA) gene sequencing are commonly used to evaluate biodegradation mechanisms.^[^ ^77^ ^]^

To correctly evaluate biodegradability, it is important to consider key biodegradation indicators that closely resemble real environmental conditions. While material‐based physical and micromolecular indicators can be used in various field tests, such as in soil or aquatic systems, environmental indicators related to the presence of microbial life may vary. For example, biogas production is a key parameter under anaerobic conditions, whereas oxygen consumption (BOD analysis) is only relevant in aerobic systems.

The diverse range of materials that are compostable, edible, bioresorbable, ecoresorbable, and biodegradable have found their way in diverse fields outside of material science, including electronics and robotics.

## 4 Robotic Deployment with Biodegradable Components

### 4.1 Deploying Robots Across Ecosystems

As researchers investigating unknown ecosystems, robots and automated devices allow scientists to explore environments that humans cannot conquer. Robots can navigate deep sea environments resisting the pressure or fly through the air and track animals from hundreds of meters above. These diverse ecosystem challenges have introduced a diverse set of robots, automated devices, or sensor networks used to understand the natural environment.^[^ ^1^, ^78^ ^]^ Just as animals have diverse adaptations allowing them to navigate these ecosystems, scientists have been inspired by plants and animals to design robots with diverse capabilities. These robots can explore remote ecosystems like the deep sea,^[^ ^79^ ^]^ collect data while tracking species or ecosystem health,^[^ ^80^ ^]^ or monitor changing conditions over time.^[^ ^81^ ^]^

The diversity of ecosystems is not only displayed in the diversity of environmental factors that determine biodegradability efficacy, but is also displayed in their diversity of location, weather, and vegetation. These factors influence the accessibility of the ecosystems, potentially making it challenging for robots to reach, enter, and traverse an ecosystem. Additionally, recovery of the robot can even be more challenging. Leaving technology behind could be a potential solution. However, once in the ecosystem, the deployment of robots bears additional challenges. Our technologies often introduce foreign materials to ecosystems, which potentially are toxic or harmful to the native wildlife. These aspects bear the question, how we should think about robot deployment.

### 4.2 When and Why Should Robots Biodegrade?

In 2015, the United Nations released the Sustainable Development Goals (SDGs) as a global call to action to combat climate change, to ensure responsible production and consumption of goods, as well as the sustainable use of oceans and terrestrial ecosystems, among other goals.^[^ ^82^ ^]^ Aligning the development of electronics and robotics systems with these goals requires prioritizing the reduction of carbon emissions and the consumption of limited resources, as well as addressing waste generation and other anthropogenic factors. Common strategies to progress toward these goals include creating extremely durable devices with long lifetimes and reusing or recycling valuable materials. The feasibility of reuse or recycling depends largely on the value of the involved materials and the possibility of recovering them after use. Furthermore, technologies must enable easy disassembly to recover primary constituents. In essence, recycling must be economically viable. An alternative sustainable approach would be to implement cradle‐to‐cradle systems, in which devices and materials are part of a regenerative design.^[^ ^83^ ^]^

If recycling is not economically viable, or if the necessary investments in recycling facilities are lacking, electronics, machines, and robots end up as technological waste, commonly referred to as e‐waste. According to the UN's Global E‐waste Monitor report, only 22.3 % of e‐waste was recycled in 2023,^[^ ^84^ ^]^ resulting in a growing amount of electronic waste. This waste often ends up in landfills, gets shipped abroad, or gets incinerated.^[^ ^85^ ^]^ Ultimately, improper handling can cause this waste to leak into our planet's ecosystems, polluting the environment and threatening our planet's health. For example, pollution through microplastics has become a significant concern, highlighting the need for responsible waste management.^[^ ^86^ ^]^ Robots or environmental analyzers that are intentionally deployed in various ecosystems will eventually fail and require recovery to prevent environmental damage or to avoid becoming permanent artifacts within the ecosystem. However, the recovery process can be significantly more complex or costly than deployment, particularly in hard‐to‐reach ecosystems. If the cost of recovering a deployed robot–taking into account localization, workforce, and retrieval logistics–exceeds the cost of the robot itself, it becomes economically impractical to recover it. Consequently, effective retrieval is only feasible for expensive robots or those deployed in easily accessible ecosystems.

In addition, dense vegetation can complicate the location and extraction of robots without causing significant disruptions.^[^ ^87^ ^]^ Some locations may pose risks to human or artificial rescuers due to extreme weather conditions, high altitudes, or because they are underwater, requiring specialized equipment and safety measures. In some cases, the financial cost of retrieval could be prohibitively high, as it might require sophisticated technology or prolonged human labor, for instance, when robots are deployed in the deep sea or underground.^[^ ^88^ ^]^

When recovery becomes impractical, using biodegradable materials is a potential route to achieve sustainable technologies. Biodegradable materials disintegrate into harmless compounds that can be fully reabsorbed by the ecosystem, thereby preventing environmental pollution and reducing waste. However, the biodegradation process depends heavily on the environmental conditions in which the materials decay. Most single‐use items, such as coffee cups and utensils, are made from paper and bioplastics like PLA, which only degrade efficiently during industrial composting.^[^ ^89^ ^]^ The conditions created in industrial composting facilities are artificially induced and predominantly unavailable in nature. Furthermore, environmental conditions vary from one ecosystem to another, which can either facilitate or hinder natural degradation. Therefore, selecting materials for biodegradation in a landfill, a forest, or in the sea, requires distinctly different material solutions.

### 4.3 Combining Biodegradable Materials and Robotic Design: ESRs

Ecoresorbable materials, as defined in Section 3, need to consider the specific environmental factors of an ecosystem, to enable decomposition without harm. The term “ecoresorbable” should, therefore, be used in combination with the specific ecosystem in which the material is degrading in, to function as a subset of the term biodegradable.^[^ ^90^ ^]^ For example, ecoresorbable in freshwater lakes specifies that a material is biodegradable under the conditions found in freshwater lakes. Hence, when developing ecoresorbable materials, they should be designed to be selectively ecoresorbable, without introducing invasive species or other anthropogenic factors that could harm the respective ecosystem.

For sustainability robots deployed in natural or artificial ecosystems, using ecoresorbable materials allows for an ecosystem‐centered approach to co‐designing materials and function. Such technology considers not only the specific characteristics of an ecosystem for operation, but also for degradation after use. This design concept goes beyond creating biodegradable robots because careful consideration of the deployment site or disposal location is necessary. For example, a robot deployed in the ocean will degrade differently than one used in a terrestrial setting or in a waste treatment facility. Therefore, we propose the term ecoresorbable sustainability robots, which emphasizes the important combination of sustainability robots and ecoresorbable materials.

Purposefully designing ESRs reduces ecological burdens and costs for waste management. This approach aligns with the sustainability goals and improves the practicability of robotic applications in hard‐to‐access regions. We proceed to evaluate the ecosystems reviewed in Section 2 to determine challenges that affect ESR deployment in diverse ecosystems.

## 5 Evaluation of Ecosystem Accessibility and Biodegradability

We assessed 21 natural and artificial ecosystems introduced in Section 2 and evaluated their capacity for the deployment of ESRs by two overarching themes: accessibility of the ecosystem and biodegradability efficacy. For each of these themes, multiple factors were selected, which are described briefly below and in more detail in the Supporting Information.

### 5.1 Ecosystem Accessibility Score

When deploying robots in ecosystems, it is critical to have easy access for deployment, maintenance, and retrieval. We categorized the accessibility of the environment to determine the feasibility of recovering the robots post‐deployment. We evaluated it by formulating an ecosystem accessibility score (EAS) as the sum of three conditions, each rated from 1 (very low) to 5 (very high), including: (i) closeness to human presence (CHP), (ii) ease to navigate terrain (ENT), and (iii) mildness of conditions (MoC). The EAS was calculated as follows:

(1) $$\text{EAS}=\left(\text{CHP}+\text{MoC}+\text{ENT}\right)-3$$

In Equation (1), we subtracted three from the sum of conditions to create an EAS range from 0 (very low accessibility) to 12 (very high accessibility). The condition scores and sums for each ecosystem are shown in **Table** **2**, and the detailed rubric for scoring each of the three conditions is included in the Table S1, Supporting Information and briefly summarized below, together with the extended scoring table for each ecosystem (Table S3, Supporting Information).

**Table 2:** Ranking of accessibility of various ecosystems. We rank ecosystems in three categories (closeness to human presence, ease to navigate terrain, and mildness of conditions) to assess their accessibility. Deep sea ecosystems are the least accessible, while agricultural fields are the most accessible. A rubric for these ranking assignments is provided in Table S1, Supporting Information. The final column lists the ecosystem accessibility score.

Ecosystems	Closeness to human presence	Ease to navigate terrain	Mildness of conditions	Total EAS
Deep sea	●○○○○	●○○○○	●○○○○	**0**
Polar	●○○○○	●○○○○	●○○○○	**0**
Tundra / alpine steppes	●○○○○	●○○○○	●●○○○	**1**
Lakes (underwater)	●●○○○	●○○○○	●●○○○	**2**
Cold deserts	●○○○○	●●●○○	●○○○○	**2**
Warm deserts	●●○○○	●●●○○	●○○○○	**3**
Semi‐deserts	●●○○○	●●●○○	●○○○○	**3**
Sea surfaces	●●○○○	●●○○○	●●○○○	**3**
Caves	●○○○○	●○○○○	●●●●●	**4**
Groundwater networks	●●○○○	●○○○○	●●●●●	**5**
Coral reefs	●●●●○	●●○○○	●●○○○	**5**
Lakes (surface)	●●●●○	●●○○○	●●●○○	**6**
Sub‐tropical forests	●●●○○	●●●○○	●●●○○	**6**
Tropical forests	●●●●○	●●●○○	●●●○○	**7**
Rivers	●●●●●	●●○○○	●●●○○	**7**
Steppes	●●●○○	●●●●○	●●●○○	**7**
Savannahs	●●●○○	●●●●○	●●●●○	**8**
Mines	●●●●●	●●●○○	●●●●○	**9**
Temperate humid grasslands	●●●●○	●●●●○	●●●●●	**10**
Temperate forests	●●●●●	●●●●○	●●●●●	**11**
Agricultural fields	●●●●●	●●●●●	●●●●●	**12**

*Closeness to human presence* refers to how proximate an ecosystem is to human settlements, infrastructure, and activity centers. Scores were assigned based on a comparison between the human footprint index^[^ ^91^ ^]^ and the ecosystem classification provided by Chengcheng et al.^[^ ^31^ ^]^ Ecosystems such as deserts, which are remote yet impacted by human activities, received a very low or low CHP ratings due to their distance from populated areas, despite some human influence. In contrast, ecosystems near urban and agricultural regions, such as temperate forests and coral reefs, received moderate to high CHP ratings due to their proximity to human populations. Ecosystems that are completely human‐made, such as mines and agricultural fields, received a very high EAS rating.

*Ease to navigate terrain* refers to how easy it is for robots to traverse a given terrain, taking into account its characteristics and assessed difficulty. Terrain characteristics consider aspects such as vegetation density and terrain condition (e.g., unstructured environments). Ecosystems with an open and easily navigable nature, such as grasslands, received a high ENT rating. Conversely, the navigation difficulty was assessed based on the need for specialized equipment, vehicles, and training. For example, technology retrieval is possible in an ecosystem with moderate ENT rating, such as the subtropical forests. Still, it requires increased effort, resulting in a lower ENT rating than that of grasslands. The ENT rating would be the lowest in challenging environments like the deep sea or caves, as accessing and navigating these ecosystems necessitates specialized vehicles, sophisticated equipment, and specific training, which all contribute to a higher level of difficulty.

*Mildness of conditions* is determined as the metric used to assess the instability of conditions within an ecosystem. Harsher conditions result in lower MoC scores. Polar and desert ecosystems have extreme temperatures that are challenging for humans. These ecosystems are also characterized by strong winds and a lack of shelter, which leads to the lowest MoC scores. Ecosystems with moderate MoC scores include subtropical forests, which are humid and warm due to heavy rainfalls and intermittent heatwaves. At the higher end of the MoC scores, the ecosystems that are most stable and have mild conditions include agricultural fields and temperate forests.

### 5.2 Biodegradability Efficacy Score

We evaluated the ability of ecosystems to reabsorb materials by formulating a biodegradability efficacy score (BES). This score evaluates both biotic factors (BFs) and abiotic factors (AFs) for each ecosystem, with a rating ranging from 1 (very low) to 5 (very high). The BES was calculated as follows:

(2) $$\text{BES}=\left(2\cdot \sum_{i=1}^{3}{\text{BF}}_{i}+\sum_{k=1}^{6}{\text{AF}}_{k}\right)-12$$

In Equation (2), the BFs (e.g., microbial abundance) and AFs (e.g., temperature and pH value) are added, whereby the BFs (three in total) receive a multiplier of two to equalize their contribution to the total score with respect to the AFs (six in total). Similar to the EAS equation (Equation 1), we subtract 12 from the overall sum to create a BES range from 0 (very low biodegradability potential) to 48 (very high biodegradability potential).

**Table** **3** details the score for each type of factor for each type of ecosystem. The detailed rubric along with each individual biotic and abiotic factors rating, provided in Tables S2, S4, and S5, Supporting Information, was determined based on values reported in previous studies^[^ ^22^, ^26^, ^46^, ^56^, ^92^, ^93^, ^94^, ^95^, ^96^, ^97^, ^98^, ^99^ ^]^ for each type of ecosystem. The published papers describe the ranges of biotic and abiotic factors and their influence, positive or negative, on biodegradability. Some factors had restricted rating options as they were binary, where a factor is only relevant by its presence or absence. These factors were rated either 1 (very low) or 5 (very high). Similarly, for factors where the literature identified three distinct conditions, we used ratings of 1 (very low), 3 (moderate), and 5 (very high) only.

**Table 3:** Ranking of how various ecosystems favor biodegradation. We rank ecosystems according to how their abiotic and biotic factors benefit the biodegradation of materials. Groundwater networks provide the least favorable conditions for biodegradation, while subtropical forests provide the most favorable conditions for biodegradation. A rubric for these ranking assignments is provided in Table S2, Supporting Information. The final column lists the biodegradability efficacy score.

Ecosystems	Abiotic factors							Biotic factors			Total BES
	Temperature	UV exposure	Oxygen availability	Mechanical forces	pH values	Soil properties	Salinity	Nutrient amounts	Microbial abundance	Water availability	
Groundwater networks	●●○○○	●○○○○	●○○○○	●●●●○	●○○○○		●○○○○	●○○○○	●○○○○	●●●●●	**12**
Mines	●○○○○	●○○○○	●●●●●	●●●●●	●●●●●	●○○○○		●○○○○	●○○○○	●○○○○	**12**
Cold deserts	●●○○○	●●●○○	●●●●●	●●●○○	●●○○○	●○○○○		●○○○○	●●●○○	●○○○○	**14**
Polar	●○○○○	●○○○○	●●●●●	●●●○○	●●○○○	●○○○○		●○○○○	●●●●●	●○○○○	**15**
Semi‐deserts	●●●○○	●●○○○	●●●●●	●●●○○	●●●○○	●○○○○		●○○○○	●●●○○	●○○○○	**15**
Warm deserts	●●●●○	●●●○○	●●●●●	●●●○○	●○○○○	●○○○○		●○○○○	●●●○○	●○○○○	**15**
Coral reefs	●●●○○	●○○○○	●●●●●	●●●●●	●●○○○		●○○○○	●○○○○	●○○○○	●●●●●	**19**
Caves	●●○○○	●○○○○	●●●●●	●●○○○	●●●●●	●○○○○		●○○○○	●●●○○	●●●●○	**20**
Deep sea	●○○○○	●○○○○	●○○○○	●●●●●	●○○○○		●○○○○	●●●●●	●○○○○	●●●●●	**20**
Steppes	●○○○○	●○○○○	●●●●●	●●●○○	●○○○○	●○○○○		●●●●●	●●●●●	●○○○○	**22**
Tundra / alpine steppes	●○○○○	●○○○○	●●●●●	●●●○○	●●●●●	●●●○○		●●●●●	●●●○○	●○○○○	**24**
Temperate humid grasslands	●○○○○	●●○○○	●●●●●	●●●○○	●○○○○	●●●○○		●●●●●	●●●●●	●○○○○	**25**
Sea surfaces	●●●○○	●●●●●	●●●●●	●●●●●	●●●○○		●○○○○	●○○○○	●●●●●	●●●●●	**32**
Lakes (underwater)	●○○○○	●○○○○	●○○○○	●●●●○	●●○○○		●●●●●	●●●●●	●●●●●	●●●●●	**32**
Lakes (surface)	●●○○○	●●●●●	●●●●●	●●●○○	●●○○○		●●●●●	●○○○○	●●●●●	●●●●●	**32**
Rivers	●●○○○	●●●●●	●●●●●	●●●○○	●●○○○		●●●●●	●○○○○	●●●●●	●●●●●	**32**
Agricultural fields	●●●○○	●●●●○	●●●●●	●●○○○	●○○○○	●●●○○		●●●●●	●●●●●	●●●●●	**36**
Tropical forests	●●●○○	●●○○○	●●●●●	●●○○○	●●●○○	●●●○○		●●●●●	●●●●●	●●●●●	**36**
Savannahs	●●●○○	●●●●●	●●●●●	●●●●○	●○○○○	●●●○○		●●●●●	●●●●●	●●●●●	**39**
Temperate forests	●●○○○	●●●●●	●●●●●	●●●○○	●●●○○	●●●○○		●●●●●	●●●●●	●●●●●	**39**
Sub‐tropical forests	●●○○○	●●●●○	●●●●●	●●●○○	●●●●○	●●●○○		●●●●●	●●●●●	●●●●●	**39**

*BFs* were assessed as nutrient amounts (BF_1_),^[^ ^36^, ^100^, ^101^, ^102^, ^103^, ^104^, ^105^, ^106^, ^107^, ^108^, ^109^, ^110^, ^111^, ^112^, ^113^, ^114^, ^115^, ^116^, ^117^, ^118^, ^119^, ^120^, ^121^, ^122^, ^123^ ^]^ microbial abundance (BF_2_),^[^ ^26^, ^39^, ^100^, ^102^, ^109^, ^114^, ^116^, ^120^, ^121^, ^124^, ^125^, ^126^, ^127^, ^128^, ^129^, ^130^, ^131^, ^132^, ^133^, ^134^, ^135^, ^136^, ^137^ ^]^ and water availability in each type of ecosystem (BF_3_),^[^ ^104^, ^109^, ^115^, ^123^, ^138^, ^139^, ^140^, ^141^, ^142^, ^143^, ^144^, ^145^, ^146^, ^147^ ^]^ which all are critical to biodegradability of materials. Ecosystems with a low nutrient availability were rated 1, while those with high nutrient availability were rated 5. The assessment of this factor was related to the nutrient needs of microorganisms present in the ecosystem. For example, a high amount of nutrients would facilitate the growth of microorganisms.^[^ ^148^, ^149^ ^]^ As the type of microbial abundance affects the degradation rates of materials,^[^ ^46^ ^]^ we rated ecosystems with dominant anaerobic bacterial populations with 1, ecosystems with facultative microorganisms with 3, and ecosystems with predominant aerobic microorganisms with 5. Water availability or moisture present in an ecosystem was scored according to the average amount of annual precipitation reported by Dlamini et al.^[^ ^94^ ^]^ As a high amount of precipitation (>1000 mm) indicates ideal conditions for microorganism growth and reproduction, we rated such conditions with 5. An annual precipitation of less than 600 mm would be unfavorable for biodegradability and was rated 1. Intermediate ranges were equally divided for ratings 2–4, with detailed ranges provided in the Supporting Information. This type of partition will also benefit future assessments, when climate change alters the value of this or any other discussed factor (biotic or abiotic).

*AFs* that were assessed for all types of ecosystems include temperature (AF_1_),^[^ ^35^, ^36^, ^38^, ^115^, ^123^, ^137^, ^139^, ^140^, ^141^, ^142^, ^145^, ^146^, ^150^, ^151^, ^152^, ^153^, ^154^, ^155^, ^156^, ^157^, ^158^, ^159^ ^]^ UV exposure (AF_2_),^[^ ^38^, ^160^, ^161^, ^162^, ^163^, ^164^, ^165^, ^166^, ^167^, ^168^, ^169^ ^]^ oxygen availability (AF_3_),^[^ ^26^, ^34^, ^170^, ^171^, ^172^ ^]^ mechanical forces (AF_4_),^[^ ^109^, ^112^, ^115^, ^152^, ^165^, ^172^, ^173^, ^174^, ^175^, ^176^ ^]^ and pH values (AF_5_).^[^ ^26^, ^100^, ^102^, ^103^, ^104^, ^109^, ^114^, ^115^, ^116^, ^117^, ^122^, ^123^, ^137^, ^153^, ^159^, ^177^, ^178^, ^179^, ^180^, ^181^ ^]^ Additionally, a sixth rating was used for either terrestrial or aquatic ecosystems to assess soil type (AF_6_),^[^ ^109^, ^112^, ^115^, ^122^, ^123^, ^125^, ^132^, ^142^, ^145^, ^174^, ^182^, ^183^, ^184^, ^185^ ^]^ or salinity (AF_6_).^[^ ^36^, ^172^, ^186^ ^]^ Higher temperatures facilitate thermal degradation and hydrolysis processes.^[^ ^187^ ^]^ Average temperatures lower than 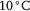 were rated 1, and an additional point was added for every 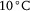 increase, ending with a score of 5 for temperatures higher than 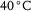. Similarly, UV radiation contributes to thermal degradation. To measure its impact, a rating scale was created. Low UV index ranges (i.e., 0 to 5) are assigned a score of 1, while an index above 10 receives a score of 5. Intermediate values are then rated with scores ranging from 2 to 4. For oxygen availability, conditions were rated as 1 if they were anaerobic and a score of 5 if they were aerobic. For mechanical forces, ecosystems with no mechanical loads received a score of 1; a score of 2 was given to ecosystems with sporadic mechanical loads, such as wind; a score of 3 was given to ecosystems with constant mechanical loads, such as tides or currents in aquatic environments; a score of 4 was given to ecosystems with sporadic multiple mechanical forces, such as fires mixed with winds; and a score of 5 was given to ecosystems with a constant presence of multiple mechanical forces, such as constant currents or high pressures in the deep sea.

The more alkaline or acidic the conditions become, the better they are for degradation processes.^[^ ^188^ ^]^ Ecosystems with neutral pH conditions (between 6 and 8) were given a score of 1. Ecosystems that are highly alkaline (below 5) or acidic (above 9) were given a score of 5, and intermediate values were distributed between these ranges. The final condition is dependent on the type of ecosystem. For terrestrial ecosystems, we evaluated the soil types, rating soils with a high presence of sand or silt with 1, soils consisting of a mixture of sand, silt, and clay with 3, and soils consisting mostly of clay with 5. For aquatic ecosystems, we evaluated salinity, rating conditions with high amounts of mineral salts with 1, conditions with a moderate presence of mineral salts with 3, and conditions with low amounts with 5, as higher amounts of salinity hinder biodegradation.

### 5.3 Categorization of Ecosystems with Respect to Biodegradability and Accessibility

**Figure 3:**
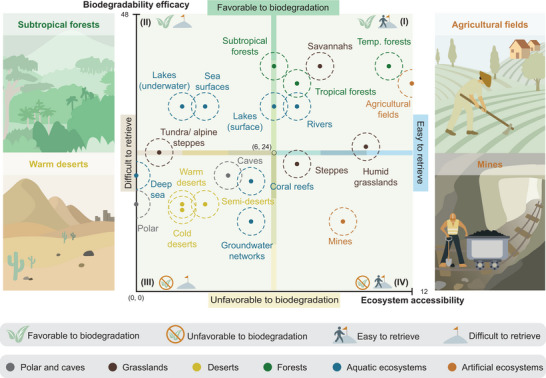
Categorization of ecosystems based on their accessibility and biodegradability efficacy. Based on our ranking of ecosystems, we create a map that categorizes ecosystems into four quadrants, providing strategies for robot design according to their end‐of‐life scenario. (I) The first quadrant includes ecosystems that are both favorable to biodegradation and easy to retrieve robots. Both retrievable and biodegradable technologies can be applied. (II) The second quadrant includes ecosystems that are favorable to biodegradation but difficult to retrieve robots. Therefore, biodegradable robots are preferred in these ecosystems. (III) The third quadrant includes ecosystems that are unfavorable to biodegradation and difficult to retrieve robots. For these ecosystems, a hybrid strategy combining retrievable technology and biodegradation may be feasible. However, increased difficulties for both retrieval and biodegradation have to be expected. (IV) The fourth quadrant includes ecosystems that are unfavorable to biodegradation but easy to retrieve robots. Therefore, retrievable robots are preferred in these ecosystems.

To understand the influence of global natural and artificial ecosystems on the deployment of ESRs we compare the ecosystems' biodegradability efficacy with their accessibility by plotting EAS versus BES and visualizing the resulting distribution in **Figure** **3**. The EAS is derived entirely from qualitative assessments, as we have established specific categories that we consider essential for evaluating the accessibility of a given type of ecosystem. Conversely, the BES is derived from a quantitative assessment, as our evaluations and categorizations are based on established literature cited in this review. Both ratings contain inherent errors, either through our own evaluations or within reported data ranges, which we aimed to illustrate visually. In the case of the EAS, the ratings for each type of ecosystem were generated based on our evaluations, which introduces a potential for error. Conversely, for the BES, it is important to recognize that our assessment represents a singular snapshot of a specific type of ecosystem at a particular point in time. Consequently, we have incorporated a ±5% margin of error in both scores, which is represented by the dashed lines around each ecosystem's score (Figure 3).

For robots deployed into an ecosystem, we can derive strategies for an end‐of‐life strategies that are based on this classification of biodegradability and accessibility. We group ecosystems into the four quadrants (I–IV) with the ecosystem accessibility score evenly distributed from 0 to 12 along the horizontal axis and the biodegradability efficacy score evenly distributed from 0 to 48 along the vertical axis. The intersection point of the axes (6, 24) is defined by the mean values of the two scores, creating the boundaries of the four quadrants.

- *(Quadrant I) Favorable to biodegradation (24 ⩽ BES ⩽ 48) and easy to retrieve (6 ⩽ EAS ⩽ 12)*. In such environments, recoverable robots can be used if there is a retrieval strategy is available that does not harm the ecosystem. Alternatively, single‐use or non‐retrievable robots may undergo natural biodegradation. For example, reusable farming robots can be used in agricultural settings as they can easily be collected and repurposed for other tasks. Additionally, small‐scale robots that are difficult to retrieve can biodegrade in soil, potentially enhancing soil fertility.
- *(Quadrant II) Favorable to biodegradation (24 ⩽ BES ⩽ 48) and difficult to retrieve (0 ⩽ EAS < 6)*. In these environments, biodegradable materials are strongly recommended. On one hand, biodegradable materials can minimize the potential ecological impact on sensitive environments. On the other hand, in ecosystems like subtropical forests, the dense stratified vegetation and high annual precipitation pose a major challenge to robot retrieval.
- *(Quadrant III) Unfavorable to biodegradation (0 ⩽ BES < 24) and difficult to retrieve (0 ⩽ EAS < 6)*. High ethical standards must be applied when considering the use of robotics in such areas. If retrieval is technologically feasible, it should be prioritized. Biodegradation in these environments is more challenging; therefore, a hybrid strategy combining recovery technology and biodegradation may be a viable solution.
- *(Quadrant IV) Unfavorable to biodegradation (0 ⩽ BES < 24) and easy to retrieve (6 ⩽ EAS ⩽ 12)*. In these areas, retrievable robots can be deployed to assist workers with hazardous tasks. At the end of their operational life, these robots can be collected for reuse, recycling, or centralized treatment through methods such as industrial composting.

This categorization guides the development of sustainable robotic technologies and suggests concrete end‐of‐life scenarios for each ecosystem. For researchers developing ecoresorbable technologies, it is important to test biodegradation in realistic conditions prior to deployment to assess the biodegradation rate of the materials used. We provided a brief overview of test methods for a variety of conditions and environments in Section 3. In the following section, we provide a framework for understanding how biodegradable technology can be effectively deployed using ecosystem‐centered design for different ecosystems, aiming to build ESRs.

## 6 Framework for Ecoresorbable Sustainability Robots

In this review we constructed and propose a framework for the design and deployment of ESRs. We propose this framework as a roadmap for future researchers who are interested in developing ESRs, emphasizing that there are multiple iterative steps for their realization. We believe that this framework will help researchers developing ESRs and biodegradable robots in general to explore more ecosystem‐specific solutions. This framework is inspired by various frameworks on human‐centered design^[^ ^189^ ^]^ as well as conservation tools^[^ ^78^ ^]^ and conservation technology.^[^ ^190^ ^]^ The nine‐step framework begins with an examination of an ecosystem, followed by three primary recursive loops: the biodegradable material loop, the robotic design loop, and the ecoresorbable robotic loop. Between each of these loops, there are intermediate steps that are less recursive but more of an evaluation of a final prototype, before moving to the next loop. Starting with the ecosystem examination, we will discuss the framework in the following four subsections.

### 6.1 Ecosystem‐Centered Design

**Figure 4:**
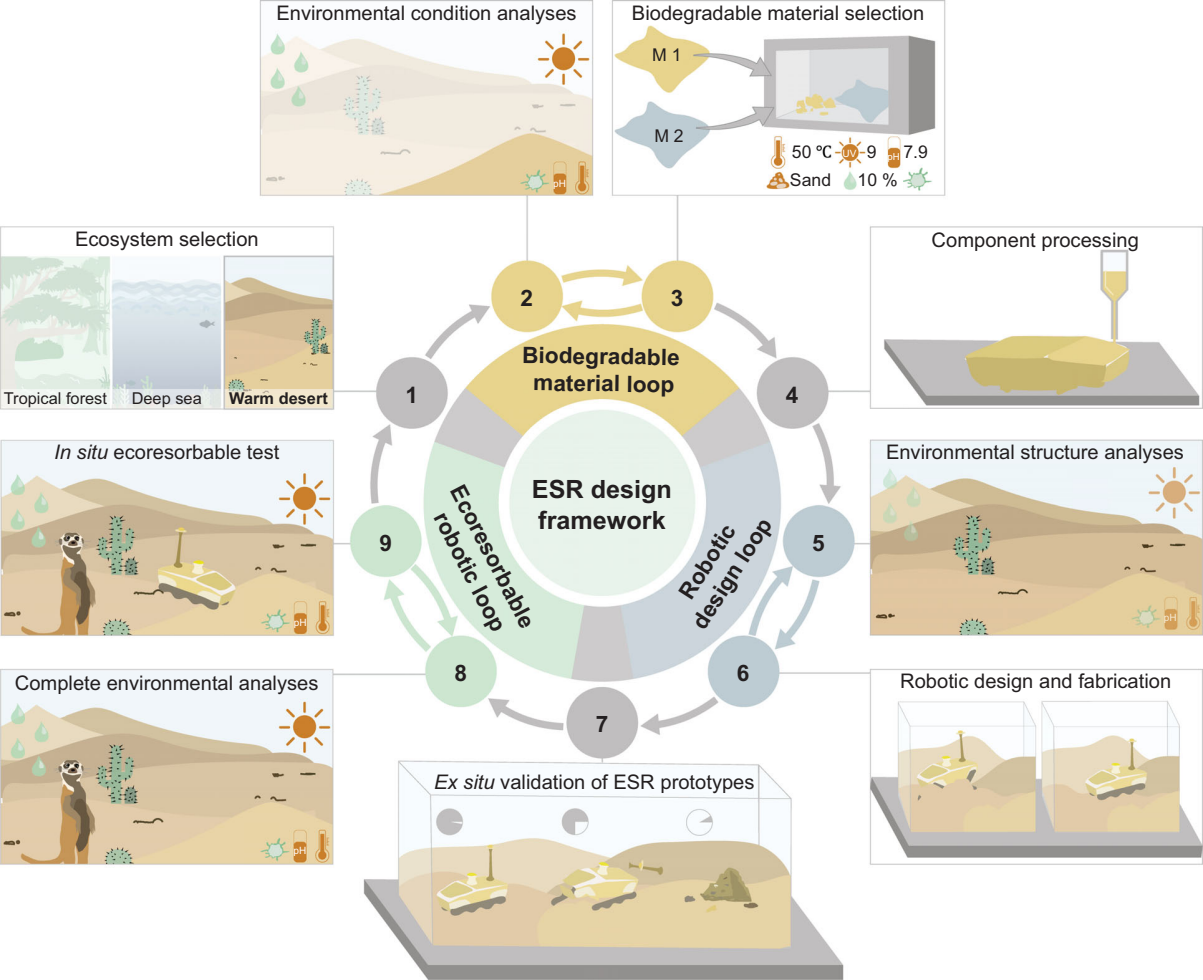
Framework for ecosystem‐centered design of ecoresorbable sustainability robots. We propose a closed cycle framework for the design of ESRs that begins with selecting an ecosystem for deployment. In this case study, we select a warm desert. The framework first starts to assess the environmental conditions and iterates through the evaluation and selection of materials through the biodegradable material loop. Then, it proceeds to building components and analyzing the structure of the ecosystem to design robots that can easily traverse the environment, which is iterated through the robotic system loop. Finally, after ex situ validation of the ESR prototype, including ecoresorbability and functionality in the simulated ecosystem, ESRs are assessed by in situ deployment in ecosystems through the ecoresorbable robotic loop. These three nested loops, starting with ecosystem selection, establish the framework for designing and implementing ESRs.

The first step in the ESR framework (**Figure** **4**) is to select an ecosystem of interest. The ecosystem selection is crucial for developing an ecosystem‐centered design of robots and materials, as it provides researchers with the necessary context. The biotic and abiotic factors of the selected ecosystem guide the development of biodegradable materials. The terrain, vegetation, and climate of the selected ecosystem inform the robot development. Each of these development steps is ecosystem‐based, which makes its selection fundamental. To illustrate the ESR framework, we selected a warm desert as an ecosystem case study. Warm deserts are a terrestrial ecosystem located in quadrant III of our categorization of ecosystems in Figure 3. Warm deserts have conditions that are unfavorable to biodegradation and have low accessibility. Deploying ESRs in such an ecosystem would be highly challenging and represents a far‐reaching goal for ESR development.

For the rest of the framework there are three loops that take inspiration from both human‐centered design and the engineering design process framework as they are iterative. Each of these presents a fundamental challenge across the disciplines of material science, chemistry, engineering, and biology. Due to the iterative nature of each loop and the challenges of material, robotic, and ecoresorbable scientific exploration, it is likely that each of these loops could produce technologies, methods, platforms, or publications. Therefore, realization of a fully ESR would likely take several years, as it involves going through three different iterative tasks. Upon selecting an ecosystem, we proceed to the biodegradable material loop.

### 6.2 Biodegradable Material Loop

The concept of the biodegradable material loop draws on the iterative nature of engineering design, involving experimentation and testing to identify suitable biodegradable materials for a given ecosystem. In the biodegradable material loop, we start with evaluating the biotic and abiotic factors of the environment. In the case of our case study, we assessed the environmental conditions of a warm desert ecosystem. Warm deserts exhibit the highest temperatures among all terrestrial ecosystems, with temperatures reaching up to 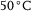.^[^ ^140^ ^]^ These high temperatures facilitate biodegradation by melting polymers and improving chain mobility. The UV radiance is also high, with a UV index of up to 9.^[^ ^163^ ^]^ The wind in warm deserts is strong, and the pH value of the soil is around 7.9.^[^ ^109^ ^]^ The annual precipitation in warm deserts is around 30–300 mm.^[^ ^140^ ^]^ Similar to semi‐deserts, most bacteria in warm deserts are aerobic, but some anaerobic bacteria, such as Bacteroidetes, are also present.^[^ ^109^, ^129^ ^]^ These biotic and abiotic factors then help us to select biodegradable materials that can function durably and do not harm specific ecosystems after their end of use.

For example, in desert applications where temperature is a dominant feature, PCL, which has a relatively low melting point (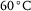), would not be a suitable choice. Instead, polymers with higher thermal stability, such as polyhydroxybutyrate (PHB) (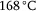
–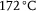), poly(butylene succinate) (PBS) (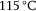), or PLA (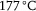
–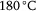), are likely to provide better mechanical and structural behavior during operation.^[^ ^191^ ^]^ Among these, PLA and PHB are known for their sensitivity to UV radiation,^[^ ^192^, ^193^ ^]^ which influences the biodegradation rate. Therefore, they would likely be more effective in a warm desert application. Depending on the intended operation time of the application, lifetime of the technology can be extended or shortened through the additives or material composites. Although it is desirable for materials to biodegrade immediately after their use, precisely controlling the degradation time remains challenging. However, some nature‐sourced biodegradable materials do not need to be readily biodegradable to be ecofriendly. For example, lignin and cellulose fibers extracted from local trees in deserts will not deteriorate the local environment in spite of their long biodegradation time.

In laboratory conditions, researchers perform controlled experiments for biodegradability utilizing the standardized techniques introduced in Table 1. Iterating through material candidates and test methods, allows testing how materials degrade under controlled conditions that are close to the biotic and abiotic factors of the chosen ecosystem (i.e., a warm desert). Once a material that effectively biodegrades is found, such as M1 shown in Figure 4, the framework can proceed to component processing. This step involves the integration of ecoresorbable materials in diverse components. These components include, but are not limited to, actuators, batteries, transmitters, sensors, receivers, micro‐controllers. These components would be combined with the biodegradable materials through different component processing techniques, such as multi‐material extrusion printing,^[^ ^194^ ^]^ to fabricate soft and stiff biodegradable materials that have been validated through the biodegradable material loop. Once the components have been processed, the framework proceeds to the robotic design loop.

### 6.3 Robotic Design Loop

In the robotic design loop, the chosen ecosystem is evaluated for its accessibility factors, which include proximity to human presence, the ease of navigating terrain, and the severity of environmental conditions. These factors will impact the robot design. The closeness to human presence gives an idea of how difficult it is to deploy and monitor a robot. The ease of navigating terrain is especially critical, as evaluation of the ecosystem's geography, vegetation, obstacles, or substrate properties will inform the mechanics of the robot's locomotion. The environmental conditions determine how resilient the robot needs to be, with respect to environmental hazards or dramatically changing conditions. When evaluating the warm desert, we observe that the environment will primarily consist of sand and be at both high and low temperatures, which will limit locomotion modes due to the challenges of traversing granular media.^[^ ^195^ ^]^ With the distance from regular human presence, tests with a tethered robot are likely to be challenging.

To effectively evaluate the ability to traverse this terrain, the fabricated components must first be tested to establish successful means of movement. For example, testing different limb configurations, such as those of quadrupedal dogs, or snake‐inspired robots, allows determination of which platform is most effective in the selected ecosystem. These locomotion modes can be first tested in tethered configurations but need to be untethered for realizing successful ESR deployment. Additionally, laboratory conditions allow simulating controlled perturbations or manufacturing arbitrary terrains. Laboratory conditions could also test how animals would perceive and/or interact with the various robotic designs. These tests are a critical start toward realization of ESRs, as laboratories allow the robot to be tested in a fixed and ex situ environment. This controlled environment minimizes any potential harm to the robot or ecosystem where it is meant to be deployed. Once the desired locomotion mode, actuators, and fabrication techniques have been tested in a controlled environment and the final prototype has been decided, we proceed out of the robotic design loop. The next step in the ESR framework is the ex situ validation of robotic prototypes, which looks to have a final validation step in deployment of ESRs (in ex situ conditions). After this step, the ESR framework proceeds to the ecoresorbable robotic loop.

### 6.4 Ecoresorbable Robotic Loop

In the ecoresorbable robotic loop, robotic prototypes are deployed to in situ environments (i.e., in the natural ecosystem they are designed for). In this loop, all factors (e.g., abiotic, biotic, and accessibility) of the ecosystem are considered. In addition to the biotic and abiotic factors that were considered in the biodegradable material loop, and the terrain that was considered in the robotic design loop, this loop considers all flora and fauna. Examples of a warm desert ecosystem include animals, such as meerkats, and plants, such as cacti, that could pose challenges for robotic traversal through animal‐robot interaction. Furthermore, in the ecoresorbable robotic loop it is critical that the ESRs do not harm the environment. Examples of harming the environment include, but are not limited to, introducing invasive species or harming native wildlife. When robots are deployed during in situ experiments researchers must consider these aspects. Our scoring of warm desert ecosystems showed they are difficult to retrieve and unfavorable to biodegradation making them one of the most challenging ecosystems for ESR deployment (Figure 3). After successful deployment of ESRs utilizing ecosystem‐centered design, the loop continues, as it is possible this robot could be deployed to additional environments. When translating an ESR to a different environment, the loop repeats; therefore, researchers must consider the new environments' biotic and abiotic factors, as well as its accessibility.

## 7 Current Developments Toward ESRs

Robots are being deployed in natural and artificial ecosystems.^[^ ^196^, ^197^, ^198^ ^]^ Given the advancements in autonomous systems, these robots are poised to become indispensable tools for mapping environmental conditions, monitoring ecosystem health, and evaluating the biodiversity of flora and fauna. Robots can measure environmental factors as they navigate an ecosystem or deploy sensors at scale to autonomously collect data, minimizing the negative impact of direct human intervention.

The first biodegradable robotic systems have been demonstrated. Based on functional materials and structures, these systems incorporate designed behaviors to achieve functionality at low structural complexity.^[^ ^199^ ^]^ However, the development of more complex systems is hindered by the limited performance of biodegradable components and their integration into robots. Yet, recent advances in hardware from biodegradable hardware, including actuation and electronics, offer promising future opportunities for the realization of ESRs.

In this section, we review the current progress toward realizing ESRs, including devices that employ biodegradable materials, ecoresorbable sensors, and actuators. **Table** **4** and Tables S6 and S7 (Supporting Information) summarize current technologies for aerial, terrestrial, and aquatic environments. Since autonomous navigation has not yet been achieved in ESRs, we first focus on the deployment of systems in aerial environments and on simple actuation tasks enabled through shape morphing. Subsequently, we discuss actuators for more complex tasks, such as manipulation and locomotion in terrestrial or aquatic environments.

**Table 4:** Research progress toward ecoresorbable sustainability robots. Summary of representative examples of ESRs and their current development stage with respect to the ESR development framework (Figure 4). A more exhaustive list of examples is included in Tables S6 and S7, Supporting Information. EC, environmentally controlled; DC, digitally controlled.

		Biodegradable material loop		Robotic design loop			Ecoresorbable robotic loop	
Technologies	Technology examples	Primary materials	Biodegradability	Actuators	Controllability	Components	In situ robotic test	In situ ecoresorbable test
Drones	Motor‐driven, edible drones [207]	Puffed rice, non‐biodegradable motors and other electronics	Partially biodegradable (no test)	Motors	DC‐remotely controlled	Actuators, batteries, transmitters, receivers, flight controllers	–	–
Gliders	Ink‐jet printed paper gliders [215]	Cellulose paper	Fully biodegradable (fully degraded after 105 days, test in moist soil)	–	EC‐autonomous	–	Deployed in artificial ecosystems	–
Sensor holders	Hygroscopic‐ally relaxing gripper [204]	Linen, balsa wood, dextrin, gelatin hydrogels	Fully biodegradable (partially degraded in water)	Humidity‐responsive materials	EC‐autonomous	Actuators	Deployed in forests	–
Wind dispersed sensors	Colorimetric fliers for environmental monitoring [211]	PLGA, cellulose, environmentally benign colorimetric reagents	Fully biodegradable (partially degraded within 84 days, norm ASTM 1998)	–	EC‐autonomous	Sensors	Deployed in artificial ecosystems	–
Shape morphing actuators	Actuators driven by temperature, light and humidity [225]	CINPs, CNFs, PLA	Fully biodegradable (almost fully degraded after 50 days, test in protease K solution)	Multi‐stimuli responsive materials	EC‐remotely controlled	Actuators	–	–
Self‐burying robots	Humidity‐responsive self‐burying robots [30]	Wood veneer	Fully biodegradable (no test)	Humidity‐responsive materials	EC‐autonomous	Actuators	Deployed in the raised bed	–
Pneumatic actuators	Omnidirectional and exteroceptive soft actuators [230]	Gelatin hydrogel, cellulose fiber	Fully biodegradable (almost fully degraded within 14 days, BOD analysis)	Fiber‐reinforced pneumatic actuators	DC‐tethered	Actuators, sensors	–	–
Electrically driven actuators	Biodegradable electro‐hydraulic grippers [229]	PLA, veget‐able‐based oils, gelatin hydrogel with NaCl	Fully biodegradable (almost fully degraded after 50 days, test in compost soil)	Electro‐hydraulic actuators	DC‐tethered	Actuators	–	–
Aquatic grippers	Aquatic hydraulic hydrogel grippers [231]	Alginate hydrogel	Fully biodegradable (fully degraded within 7 days, test in artificial marine reef aquarium)	Hydraulic actuators	Hybrid EC‐DC‐tethered	Actuators	–	–
Aquatic swimming robots	Aquatic swimming robots driven by chemical reaction [233]	Fish feed pellets, gelatin, citric acid, sodium bicarbonate, propylene glycol	Fully biodegradable (no test)	Pneumatic actuators driven by chemical reaction	EC‐autonomous	Actuators, energy supply	–	–

### 7.1 Aerial Environments

#### 7.1.1 Drones, Gliders, and Sensor Holders

Uncrewed aerial vehicles (UAVs), including quadcopter drones and propelled gliders, provide a versatile and accessible platform for environmental monitoring and research.^[^ ^1^ ^]^ Their ability to operate independently of terrain makes them attractive for navigating various ecosystems. When deployed near areas with human presence, UAVs can be easily launched and returned, enabling efficient data collection without or with minimal physical interaction with the ecosystem. In such cases, UAVs can map environmental factors remotely, minimizing the risk of ecosystem disruption.^[^ ^200^ ^]^ Although remote sensing with UAVs provides sufficient data for monitoring some wildlife, it can also cause stress to animals due to the generated noise and unfamiliarity.^[^ ^201^, ^202^ ^]^ Furthermore, UAVs are not a universal solution for all ecosystems, as they cannot yet operate in extremely cluttered or turbulent environments.

UAVs can also be used to deploy sensors at strategic locations or distribute them randomly at scale, eliminating the need for human intervention or intrusion into an environment. The collected sensor data can then be transmitted or recorded remotely, providing valuable insights into ecosystem dynamics. However, the large number of randomly deployed sensors via air increases the risk of waste being left in the ecosystem, which motivates the use of ecoresorbable materials.^[^ ^44^ ^]^ Similarly, in cases of failure of the UAV or when intentional physical interaction with the ecosystem is necessary, ecoresorbable materials become a preferable solution to prevent negative human impact on the ecosystem.

**Figure 5:**
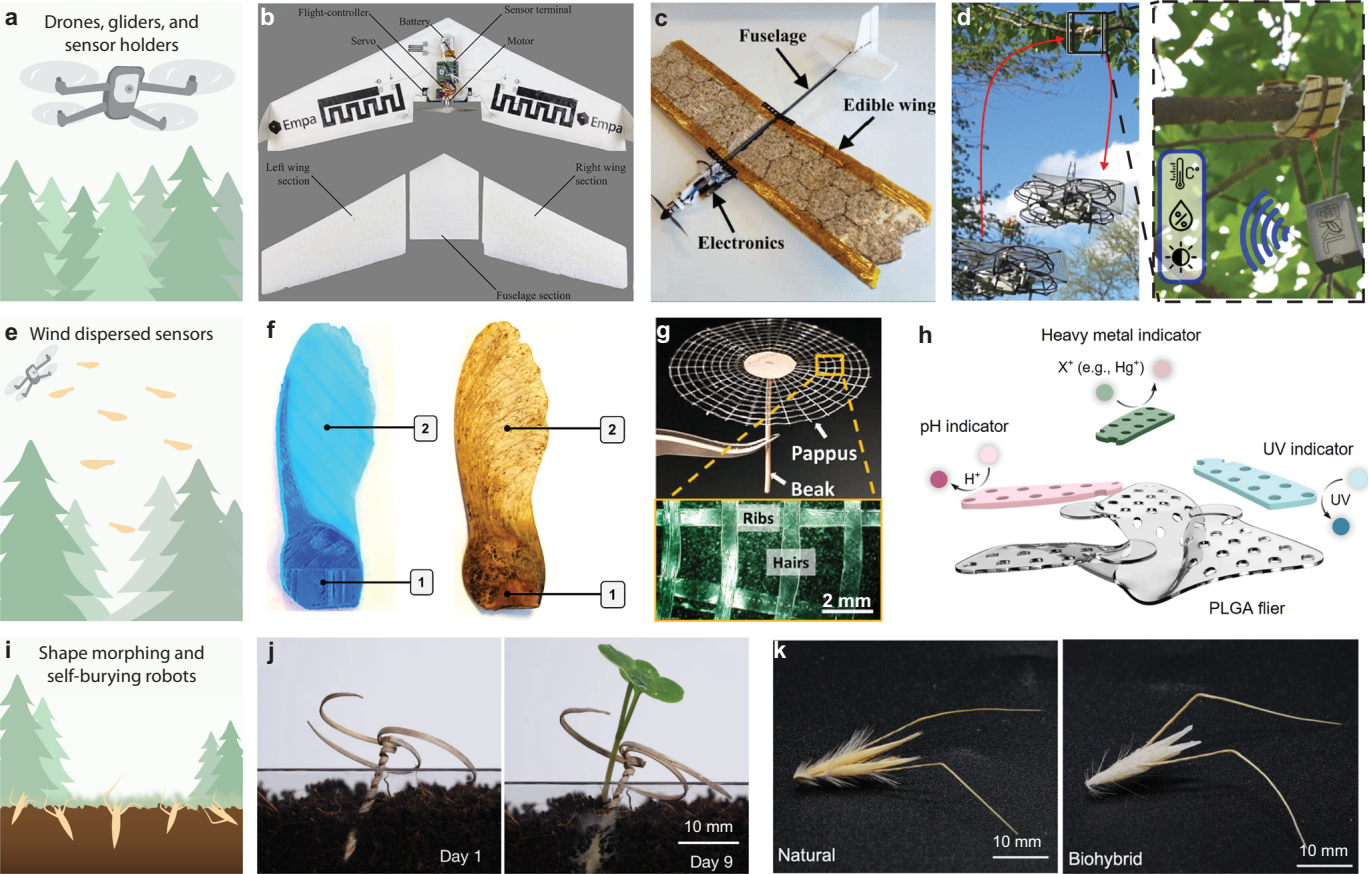
Ecoresorbable sustainability robots deployed from sky to ground. a) Schematic of an UAV in the air. Such an UAV can have onboard sensors or place sensors in strategic locations. b) A complete drone based on a cryogel with integration of electronic components. Reproduced with permission.^[^ ^208^ ^]^ c) A partially biodegradable drone with edible wings made of puffed rice. Reproduced with permission.^[^ ^207^ ^]^ d) Outdoor deployment of a biodegradable gripper through an UAV. The enlargement is a gripper wrapped around the tree branch, holding a commercial sensor. Reproduced with permission.^[^ ^204^ ^]^ e) Schematic of wind dispersed sensors descending from UAVs. UAVs can also deploy sensors randomly on the ground, benefiting from wind dispersal enlargement of coverage. f) Comparison of the artificial and natural Acer campestre seeds. Reproduced with permission.^[^ ^209^ ^]^ g) Picture of the parachute‐like flier composed of pappus and beak. The enlargement is the microscopic image of the pappus, showing ribs and hairs. Reproduced with permission.^[^ ^210^ ^]^ h) Schematic illustration of the 3D biodegradable flier with multiple colorimetric indicators including pH, UV, and heavy metal indicators. Reproduced with permission.^[^ ^211^ ^]^ i) Schematic of shape morphing actuators on the ground. j) The germination process of the seed buried by a three‐tailed hygromorphic coiling actuator. Reproduced with permission.^[^ ^30^ ^]^ k) Picture of natural and artificial wild oat (*Avena sterilis L*.) fruit consisting of two hygroscopic awns. Reproduced with permission.^[^ ^212^ ^]^

UAVs can deliver sensors or environmental analyzers to strategic locations, such as tree branches, and mount them for long‐term ecological readings (some examples are highlighted in **Figure** **5a–d**).^[^ ^203^ ^]^ This approach enables targeted, locatable measurements or mapping of an ecosystem by utilizing sensor networks.

One energy‐efficient method of sensor placement is to use a hygroscopic gripping system to mount sensors in tree canopies.^[^ ^29^ ^]^ The system releases stored elastic energy in response to changes in humidity to initiate the folding of a helical structure that wraps around tree branches. A commercial sensor integrated into the gripper can measure temperature, humidity, barometric pressure, ambient light, and sensor altitude from the deployed position. To reduce plastic waste and potential pollution, biodegradable alternatives were used to replace the materials of the gripper (Figure 5d).^[^ ^204^ ^]^ Linen textiles were used as a flexible layer, balsa wood as a stiffening layer, and prestretched gelatin‐based hydrogels as a humidity‐responsive material. A similar approach for mounting sensors on tree branches was used by Heinrich et al.^[^ ^205^ ^]^ Their hygroscopic gripper features a structure made of starch, cellulose, and a carbon‐loaded shellac ink that coils around branches as humidity changes.

When selecting materials for such applications, it is essential to consider their potential impact on the ecosystem. Animals interacting with the technology may have unintended consequences, such as birds using potentially harmful materials for nest building or food.^[^ ^206^ ^]^

If designed purposefully, robots made from edible components could be used to provide food or to enable faster biodegradation rates. For example, UAVs with edible wings from puffed rice were introduced as a deployable food source (Figure 5c).^[^ ^207^ ^]^ When deploying technology in nature, it is crucial to consider the type of food that would be introduced into the ecosystem. Mindful materials design can leverage edible structures. Sourcing materials from the deployment site can help to identify noninvasive material sets while benefiting from biodegradation.

When UAVs crash and fall to the ground, biodegradable components can also reduce the harm and pollution in natural ecosystems. Wiesemüller et al. manufactured a partially biodegradable glider,^[^ ^208^ ^]^ in which gelatin was introduced into a network of microfibrillated cellulose to form a biodegradable material with high strength for the glider's airframe (Figure 5b). An ink of carbon‐black nanoparticles was printed on the elevon of the glider to add sensing functionality and measure its deflection angle. In addition to the biodegradable body, the required conventional electronic components, including the battery, motors, and flight controllers, were incorporated into a sealed housing to avoid potential environmental contamination.

#### 7.1.2 Wind Dispersed Sensors

UAVs can be used to release sensors in the air. The sensors are then carried by the wind, taking advantage of gravity, to cover expansive areas upon reaching the ground (Figure 5e).^[^ ^213^ ^]^ To increase coverage, researchers have designed seed‐inspired structures that begin to rotate during free fall.^[^ ^214^ ^]^ Environmental conditions can be measured during flight or after landing using functional sensing materials. However, when deployed at scale, the large number and low cost of sensors could pollute the ecosystems in which they are deployed. However, Sethi et al. argue that biodegradable sensor systems would enable such large‐scale deployment without harming the environment.^[^ ^3^ ^]^

Paper is a possible material for creating low‐cost gliders through frugal design and fabrication.^[^ ^215^ ^]^ The gliders are fabricated by inkjet printing a solution of lithium chloride on paper to fold it into a 3D structure upon drying. After 15 weeks, the paper mostly disintegrates in soil without polluting the environment. The type and complexity of the involved materials in gliders and wind‐dispersed sensors largely depend on how sensor data is collected. Passive sensors can react to changes in the environment's pH, temperature, and humidity by changing their color or fluorescence intensity. The change can later be remotely monitored by another UAV or agent passing by. A glider inspired by the Alsomitra macrocarpa seed was developed for sensing the pH of rainwater and could be used in ecosystems with high precipitation.^[^ ^216^ ^]^ While flying in the rain, the humidity‐responsive glider deformed into an open state, exposing the litmus‐based pH sensor that changes color according to the acidity of the rainwater.

Plant seeds that utilize an extended flying phase to increase their range are often used as inspiration for designing artificial flyers. For example, fliers with temperature‐sensing function were fabricated via 3D printing of PLA by mimicking the shape of Acer campestre seeds (Figure 5f).^[^ ^209^ ^]^ By incorporating fluorescent lanthanide‐doped particles into the PLA matrix, the fliers can absorb near‐infrared light and emit green fluorescence. The fluorescence intensity changes with temperature, providing reliable results in the range from 268 K to 313 K (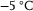 to 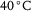) and a resolution of 0.54 K at a high signal‐to‐noise ratio. This implies the potential for operation in ecosystems with large temperature variations. Mariani et al. reported a porous, parachute‐like flyer that can detect the concentration of soil nitrate (Figure 5g).^[^ ^210^ ^]^ To this end, they mounted a commercial colorimetric nitrate indicator on the flyer using a cellulose acetate solution as an adhesive. When the flyer descended onto nitrate‐contaminated soil, the color of the indicator changed from white to violet, indicating a high level of nitrate contamination. This approach could be promising for detecting soil contamination in steppe and savannah ecosystems for grassland restoration.

Multimodal sensing was also demonstrated in wind‐dispersed fliers to gain a more comprehensive understanding of ecosystems.^[^ ^211^, ^217^ ^]^ Yoon et al. introduced a biodegradable flyer that enabled the colorimetric assessments of pH, heavy metal concentrations, ultraviolet exposure, humidity, and temperature (Figure 5h).^[^ ^211^ ^]^ The flyer included a 3D substrate composed of poly(lactic‐co‐glycolic acid) (PLGA) and colorimetric indicators primarily made of cellulose. The indicators were prepared by vacuum filtration of environmentally benign colorimetric reagents through the cellulose film. For example, the pH indicator used anthocyanin extracted from red cabbage to produce the colorimetric response across a range of pH values. With the assistance of fungi, these indicators and the PLGA substrate were partially biodegraded within 12 weeks.

A major limitation of systems that utilize color changes is their lack of memory and ability to store data. This shortcoming requires some means of remote data recording, for example, through a passing UAV. Active sensing, data storage, and transmission would require energy harvesting or storage. Although there are a few examples of batteries and energy harvesters made from transient material systems,^[^ ^218^, ^219^, ^220^, ^221^ ^]^ they are not yet efficient at the small scales that are required by wind dispersal sensors, and their multi‐material architectures make degradation more difficult in diverse environments.

### 7.2 Terrestrial Environments

#### 7.2.1 Shape Morphing and Self‐Burying Robots

In addition to sensing, changes in environmental conditions can be used to drive actuation. Inspired by plants, shape morphing structures can translate changes in humidity or temperature into motion at low complexity.^[^ ^5^, ^222^ ^]^ This behavior can be used for gripping tasks, such as when deploying sensors, or for drilling into soil as illustrated in Figure 5i. Such devices can therefore perform tasks after deployment, receiving stimulus and control from the environment itself.

For example, hygroscopic structures leverage the swelling of materials at high humidity to produce shape morphing and motion, making them suitable for ecosystems with high precipitation, like forests and savannahs. Such materials can be made from entirely biosourced and biodegradable materials. Zhao et al. demonstrated a pollen‐paper‐based actuator that can mimic the blooming of the Michelia flower and perform self‐propelled motion that is hygroscopically driven.^[^ ^223^ ^]^ The pollen paper was derived from natural sunflower pollen grains by using a two‐step soap‐making process. To increase the speed at which hygroscopic structures react to humidity changes, Sun et al. used sodium alginate hydrogel, polyvinyl alcohol (PVA), and carbon nano‐powder to prepare a biodegradable humidity‐responsive film.^[^ ^224^ ^]^ Sodium alginate was employed due to its high hygroscopicity, and the addition of PVA and carbon powder highly increased the bending angle and speed of the film. When exposed to a relative humidity of 90 %, the film can achieve a maximum bending angle of 248 ° within a few seconds.

Structures responding to a single environmental factor have limited versatility, motivating the development of multi‐stimuli‐responsive actuators. Taking advantage of the capability of photothermal conversion of cuttlefish ink nanoparticles (CINPs), hygroscopic sensitivity of cellulose nanofibers (CNFs), and the thermal expansion of PLA, Chen et al. developed biodegradable films that exhibited reversible deformation under near‐infrared light, humidity, and temperature stimuli.^[^ ^225^ ^]^ Altering the design of such functional structures during assembly allows the realization of different applications, such as grasping and lifting objects.

For the examples above, continuous actuation requires reversible changes in environmental conditions, which repeat frequently and may only be feasible in ecosystems with large diurnal fluctuations. To address this limitation, recent research has explored ways to stimulate continuous actuation through a single change in environmental conditions. Luo et al. designed a hygromorphic coiling actuator made from wood veneer that can carry a seed and self‐bury into soil (Figure 5j).^[^ ^30^ ^]^ It exhibited a higher success rate of soil drilling than the natural Erodium seeds, improving the effectiveness of aerial seeding, which shows the potential application of reforestation and grassland restoration. In a similar approach, Cecchini et al. leveraged 4D printing technology to replicate the structure and dynamic morphological changes of Geraniaceae seeds to achieve a self‐burying structure.^[^ ^226^ ^]^ The artificial seed encompassed a lever made from PCL, acting as the anchoring point, and a hygroscopically morphing fiber composed of a polyethylene oxide (PEO) shell and a cellulose nanocrystals (CNCs) core. Using two stimulus‐responsive structures enables a combination of drilling and translation motion. Inspired by Avena fruits, Fiorello et al. leveraged two hygroscopic awns to integrate a bending tail and a twisting beam (Figure 5k).^[^ ^212^ ^]^ The entanglement between the two awns enabled the structure to carry a seed and move on the ground before penetrating into the soil.

Despite the potential of hygroscopic robots for large‐area seeding, their reliance on generating motion from environmental stimulus hinders them from being used in ecosystems with low precipitation or harsh conditions, such as deserts or polar regions. In such ecosystems, fluidically driven or electrically driven actuators have the potential to work across a large range of conditions.^[^ ^28^, ^227^ ^]^

#### 7.2.2 Actuation on Land

**Figure 6:**
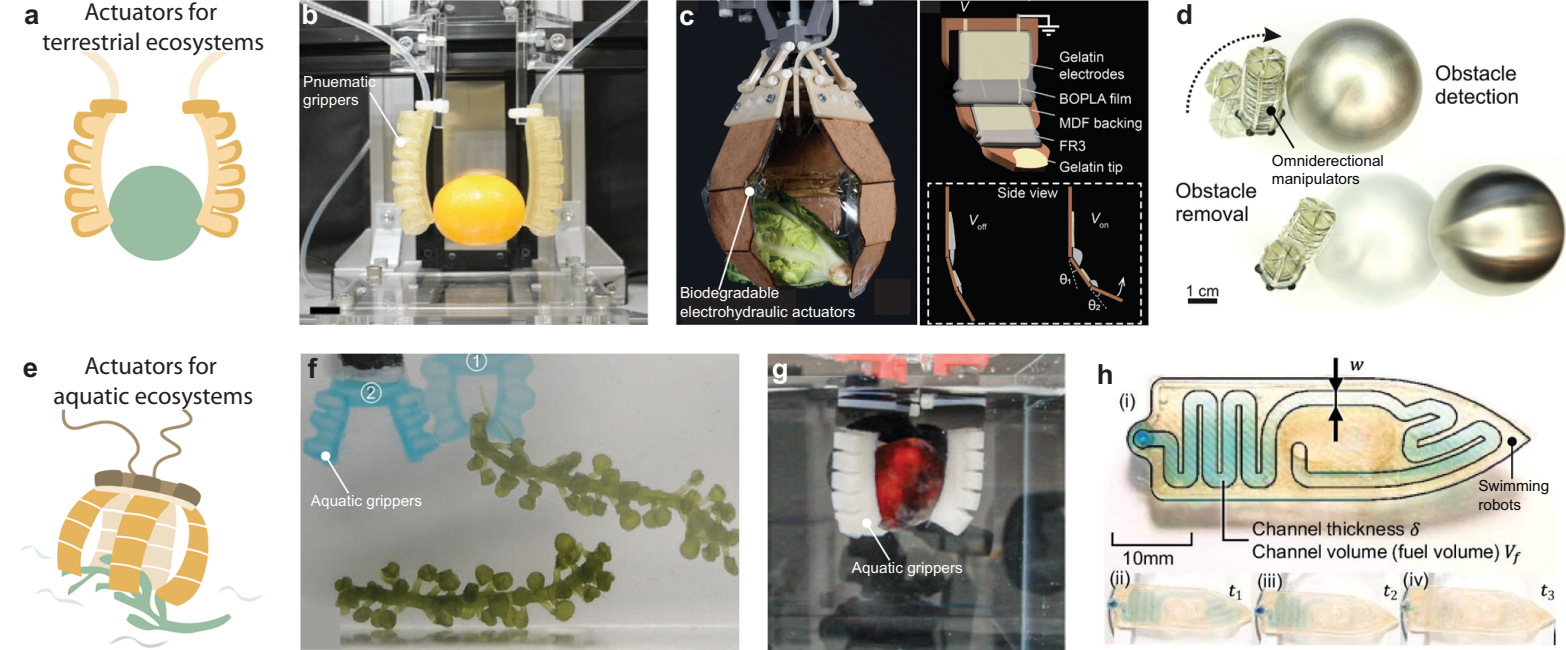
Biodegradable soft robotic systems for terrestrial and aquatic ecosystems. a) Schematic of a tethered gripper for manipulating fragile objects in terrestrial ecosystems. b) A 3D printed gripper from nanocellulose reinforced gelatin composites. Reproduced with permission.^[^ ^228^ ^]^ c) Picture of a biodegradable electro‐hydraulic gripper grasping a lettuce head (left). Schematic illustration of the structure and components of one gripper unit (right). Reproduced with permission.^[^ ^229^ ^]^ d) Biogel‐based pneumatic actuators integrated with waveguide sensors enabled the detection and removal of obstacles. Reproduced with permission.^[^ ^230^ ^]^ e) Schematic of biodegradable aquatic robots, capable of manipulation. f) A calcium alginate hydrogel gripper manipulated the sea grape underwater. Reproduced with permission.^[^ ^231^ ^]^ (g) An edible and waterproof gripper lifted a plum underwater. Reproduced with permission.^[^ ^232^ ^]^ (h) Schematic illustration of an edible robot capable of swimming on the water surface using the Marangoni effect. Reproduced with permission.^[^ ^233^ ^]^

Navigating and interacting with terrestrial ecosystems demands more complex machines, which cannot be achieved solely through shape morphing materials. Locomotion in unstructured terrain requires controllable actuation, driven by on‐demand stimulus. Although current soft robots often have relatively low complexity compared to their hard counterparts, they are rapidly evolving and demonstrating potential in diverse applications.^[^ ^12^ ^]^ Soft actuators have been widely used and studied in the context of grippers for manipulating fragile objects, as well as enabling locomotion such as crawling, inching, and walking, as shown in **Figure** **6a–d**. Biodegradable solutions for soft actuation are still scarce, but innovation in biodegradable materials has enabled a few promising examples.^[^ ^13^ ^]^

##### Pneumatic Actuators

Among soft actuators, pneumatic actuators are the most commonly used in soft robots due to their ability to generate sufficiently large forces, ease of fabrication, and structural simplicity. They typically consist of elastomers that inflate in controlled patterns, due to engineered mechanical constraints. The simplicity of these material systems makes pneumatic actuators an attractive platform for enabling ecoresorbable machines. Early pioneering works introduced biodegradable actuators based on a PneuNet architecture, although with limited durability.^[^ ^234^ ^]^

Baumgartner and Hartmann et al. overcame durability limitations by developing resilient gelatin‐based biogels, resulting in fiber‐reinforced pneumatic actuators that were actuated over 300 000 times.^[^ ^235^ ^]^ This gelatin‐based biogel is readily biodegradable and can also be 3D printed to allow for more complex shapes with increased functionalities (Figure 6b).^[^ ^228^, ^230^ ^]^ Aiming for sustainable manufacturing, Heiden et al. utilized egg albumen as biodegradable support material during printing of gelatin‐based biogels to make joint‐like actuators driven by negative pressure.^[^ ^236^ ^]^

While gelatin‐based biogels typically start degrading when exposed to media containing microorganisms, triggers for biodegradation can be built into actuators themselves. Inspired by the germination of ophiocordyceps, Yamada et al. embedded seeds within an open‐cell cellulose foam and encapsulated the foam with a biodegradable thin‐film, thereby isolating the seeds from fresh air.^[^ ^237^ ^]^ Failure of the outer shell exposed the seeds and allowed the germination, which triggered the biodegradation of the actuator at its end of life.

The use of biomaterials can also enable self‐healing of actuators, adding functionality that is difficult to achieve in metal‐based structures. Cornella et al. developed a castor‐oil‐based self‐healing elastomer for pneumatic joints.^[^ ^238^ ^]^ Although this material is not biodegradable in its crosslinked form, incorporating self‐healing capabilities can enable more durable devices. For achieving biodegradable self‐healing materials, Pena‐Francesch et al. developed materials from biosynthetic squid protein.^[^ ^239^ ^]^ Two protein membranes were combined to form a soft pneumatic actuator that can be degraded with acid stimulation owing to the non‐covalently crosslinked network structure of the protein.

Combining actuation with sensing in biodegradable systems is a first step toward creating more complex machines. Heiden and Preninger et al. integrated waveguide sensor networks on pneumatic actuators.^[^ ^230^ ^]^ As shown in Figure 6d, the sensorized actuators were capable of omnidirectional motion while the integrated waveguides provided information on their deformation states and on external touch events, endowing them with proprioceptive and exteroceptive functionalities. Ionic conductive gelatin‐based biogels can also be utilized as strain sensors by measuring their impedance.^[^ ^240^, ^241^ ^]^ Such sensors were integrated on a soft robotic arm from foldable cellulose films. The system achieved multidirectional bending as well as linear construction through the use of three motor‐tendon actuators to drive the soft robot, but architectures driven by pneumatics are possible as well.^[^ ^242^ ^]^

Although promising works have been conducted on biodegradable pneumatic actuators, this technology is often tethered to bulky (non‐degradable) pressure pumps, which limit their applications in natural environments. Electrically driven actuators, on the other hand, require significantly smaller power supplies and control systems, which can be made more lightweight.

##### Electrically Driven Actuators

Compared to pneumatic actuators, soft electrostatic actuators, such as dielectric elastomer actuators (DEAs) or electrohydraulic actuators, offer several advantages, including high actuation speeds, increased power‐to‐weight ratios, and electronic control.^[^ ^243^ ^]^ As a result, they are promising for fully untethered systems that can explore natural ecosystems autonomously.

Soft electrostatic actuators are deformable capacitors that typically utilize dielectrics as charge‐separating layers. The typically high voltages (>1 kV) to drive them make the use of biomaterials challenging. This operating condition requires materials with low conductivity and high breakdown strength, excluding hydrophilic polymers, such as gelatin‐based hydrogels. Takai et al. used natural rubber as the dielectric membrane and ionically conductive gelatin‐gels as electrodes to fabricate DEAs.^[^ ^244^ ^]^ Although natural rubber is bio‐sourced, it is hard to biodegrade when chemically crosslinked, demonstrating the need for more biodegradable elastomers with good dielectric properties.

Alternatively, soft electrohydraulic actuators can be made from flexible polymer thin‐films filled with a liquid dielectric. Rumley and Preninger et al. evaluated a fully biodegradable materials set for electrohydraulic actuators, comprising biaxially‐oriented PLA films as solid dielectric, vegetable‐based oils as liquid dielectric, and gelatin‐based biogels as electrodes (Figure 6c).^[^ ^229^ ^]^ In combination with sheets of wood as stiffening layers, they demonstrated a compostable soft robotic gripper. The gripper was demonstrated as an end‐effector on a conventional robot arm to handle waste. In such cases, the gripper can be disposed of after its end of life without generating additional waste. Another option for the solid dielectric of electrohydraulic actuators is a composite of PLA and poly(butylene adipate‐co‐terephthalate) (PBAT), which was used in combination with soybean oil as a liquid dielectric.^[^ ^245^ ^]^ The above‐mentioned biopolymers for the solid dielectric decompose within about one month in soil during industrial composting at a temperature of 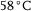.

The need for electronic control and power supplies makes electrostatically driven actuation challenging to realize in fully biodegradable systems. As a first step, a hybrid strategy could be employed, where control circuits are recovered from the deployment site. The relatively lower mass of the control electronics compared to the rest of the robot body would make it easier to recover smaller parts of robots using a UAV, mitigating the negative effects on ecosystems. Biodegradation can facilitate easier separation of control electronics from the rest of the robotic body. Such a strategy would also be effective in aquatic environments, where parts could float to the water surface, facilitating easy recovery.^[^ ^246^ ^]^

### 7.3 Aquatic Environments

#### 7.3.1 Underwater Grippers

Exploration and monitoring of aquatic ecosystems, particularly freshwater ecosystems, is crucial for maintaining precious resources and biodiversity. When navigating in bodies of water, robots can benefit from predictable, open environments and support of their weights through buoyancy. However, locomotion can become challenging in dynamic (rivers), extreme (deep sea), or cluttered (lake shores or ponds) environments. So far, field research has predominantly relied on hard and bulky, yet resilient, robots. However, similar to UAVs, propulsion driven by propellers can be noisy in water, disturbing flora and fauna in aquatic ecosystems.^[^ ^247^ ^]^ Biomimetic swimming robots better blend into such ecosystems due to locomotion strategies that are silent and biomimetic, often enabled by soft materials.^[^ ^28^, ^248^, ^249^ ^]^ Soft materials also benefit from being incompressible, providing a cost‐effective solution for deployment in the deep sea.^[^ ^250^, ^251^ ^]^ This shift from metal‐based to polymer‐based swimming robots has huge potential for swimming robots. Despite their potential, aquatic ESRs are still in their infancy and have only been realized at low complexity (Figure 6e–h). Challenging for their realization is that biodegradation in aquatic ecosystems is often more challenging than in terrestrial ecosystems.

Biodegradable alternatives (e.g., gelatin‐based hydrogels) to silicone elastomers often dissolve too quickly in water. Conversely, bioplastics, such as PLA, PBAT, poly(butylene succinate) (PBS), polyhydroxyalkanoates (PHA), and PCL, which are candidates to replace commodity plastics, can only effectively degrade during industrial composting but barely biodegrade in water, generating microplastic pollution.^[^ ^26^ ^]^ The biodegradability of these polymers varies significantly depending on the aquatic environment. For example, Wang et al.^[^ ^252^ ^]^ investigated the biodegradability of PLA, PBAT, PBS, and PCL in various bodies of water, including natural seawater, static seawater, static river water, sterilized distilled water, sterilized natural seawater, and lab‐prepared artificial seawater. In this study, only PCL showed significant degradation (32 %) in natural seawater, while the other polymers exhibited negligible degradation in any of the tested types of water. Another study^[^ ^253^ ^]^ examined the biodegradability of PLGA, PCL, PLA, and PHB in artificial seawater and freshwater over one year. In the study, PLGA completely degraded, PHB showed limited degradation (8.5 %), and PCL did not degrade at all. These findings highlight the significant influence of specific aquatic environments on the biodegradability of polymers.

For soft materials for aquatic environments, hydrogels are a promising material class for use in soft robots or as functional materials. Sun et al. produced water‐tight calcium alginate hydrogel actuators, which are biodegradable, edible by marine organisms, and exhibit shape and stiffness morphing capabilities.^[^ ^231^ ^]^ The authors used freeform reversible embedding of suspended hydrogels (FRESH) printing, a technique for embedded 3D printing, to fabricate fluidically driven hydrogel actuators at a small scale. The versatile fabrication process allowed printing of bending, twisting, and linear actuators and the fabrication of a soft gripper (Figure 6f). Degradation of the material was tested in a marine reef aquarium at ambient conditions (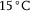), in which it degraded within the first week of incubation. Notably, the calcium alginate was sourced from brown seaweed, demonstrating an example of how materials can be sourced from a possible deployment site of the robot, reducing the risk of introducing invasive species to an ecosystem.

Kanno et al. developed water‐resistant soft actuators and a gripper for underwater applications using konjac glucomannan (KGM), a plant‐based, edible material^[^ ^232^ ^]^ (Figure 6g). KGM has practical mechanical properties, such as a stretchability of 67 % strain, with a Young's modulus of 77 kPa. The material is stable in distilled water for prolonged times (> 4 months) but can degrade in seawater within 14 days, providing usability of a few days in marine environments. This stability enabled a soft gripper that was assembled from three PneuNet actuators and operated underwater for more than 3 days.

#### 7.3.2 Swimming Robots

On the water surface, locomotion using the Marangoni effect, where a local change in surface tension using a drop of surfactant ejection is exploited for propulsion, enables untethered movement for small‐scale robots. Zhang et al. described the fabrication of fully biodegradable and edible self‐propelled aquatic robots made of freeze‐dried fish food and a binder, gelatin^[^ ^233^ ^]^ (Figure 6h). Propylene glycol was selected as a surfactant due to its low toxicity index and high solubility to allow prolonged motion. The ejection of the surfactant was driven by gas generation in a reaction chamber, which is sealed by a water‐permeable gelatin membrane. To produce the gas, the powder chemicals, sodium bicarbonate, and citric acid were used, which are not harmful to aquatic fauna. This centimeter‐scale aquatic robot achieved an average speed of 0.052 m s^−1^. Such a system might find application in the deployment of sensors on the surface of lakes, bringing a sensor to a desired location.

Beyond actuation, functional materials can aid in cleaning water from microplastic pollution. Giuolang et al. prepared algae‐based bio‐hybrid micro robots (DPA‐algae) to remove nanoparticles from the water environment.^[^ ^254^ ^]^ It is explained that active bio‐hybrid micro robots have self‐propulsion and surface functionalization to efficiently remove contaminants from water. Algae were modified by a click chemical reaction (cycloaddition reaction between DPA and DBCO) for the rapid capture of nano‐plastic particles in water samples. Compared with unmodified algae, DPA‐algae robots' removal efficiency increased by 83 %, proving that this strategy can be used as a self‐driven algae‐based micro robot to remove nano‐plastics from water.

These different technologies show promise for the development of ESRs, but there are currently no technologies that complete the full ESR design process. In the next section, we discuss some challenges of ESR development and promising research directions that could interface with ESRs.

## 8 Challenges and Opportunities in the Development of ESRs

The development of ESRs for the deployment in natural ecosystems faces numerous challenges, including materials, technologies, and ecosystem‐specific difficulties, as well as testing and validation prior to deployment. To our knowledge, current literature (Table 4; Tables S6 and S7, Supporting Information) does not have any in situ ecoresorbable tests as proposed as the final step in the ESR design framework on Figure 4. Therefore, we highlight some of the potential challenges toward the realization of ESRs.

### 8.1 Materials Challenges

Materials for ESRs face two primary challenges: 1) they should be sufficiently durable in the deployed ecosystem and 2) they should biodegrade in the deployed ecosystem, ideally when triggered. While this combination of performance and sustainability is difficult to achieve, it is possible by addressing intermediate challenges along the way. A significant challenge is to improve the stability of biodegradable materials in varied conditions. So far, materials have mostly been developed and tested for constant, controlled environments, such as indoors or in artificial ecosystems.^[^ ^224^, ^229^, ^245^, ^255^ ^]^ In natural ecosystems; however, temperature, humidity, and UV radiation are frequently changing, requiring materials to be durable in a larger range of conditions. Thermal stability needs to be increased to allow for reliable operation in warm weather or when heated by the sun. In terrestrial ecosystems with high precipitation or in aquatic ecosystems, materials must be sufficiently stable when in contact with water. Many of the current materials and components for robots are designed for terrestrial ecosystems, but not for aquatic ecosystems.^[^ ^210^, ^225^ ^]^ Regardless of the ecosystem, materials and robot components must be durable enough to allow prolonged operation.^[^ ^14^ ^]^ Testing materials for fatigue under repeated mechanical load or during actuation and optimizing them for this purpose will be essential for their widespread adoption.

The most ideal biodegradable material would remain stable during operation, but then degrades rapidly once the robot is no longer in use. Controlling the mechanism and timing of the degradation poses a significant challenge that can be addressed through various strategies. For example, Huang et al. presented a method of embedding enzymes within biodegradable polyesters, such as PBS, poly(butylene succinate‐co‐adipate) (PBSA) or PCL.^[^ ^256^ ^]^ The enzymes enable and accelerate biodegradation, potentially allowing decomposition independent of the microbes that are present in the respective ecosystem. Consequently, this embedded enzyme approach allows a broader range of materials to be used in diverse ecosystems. While promising, this embedding method has only been validated in buffer solutions and at a temperature of 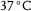, so it is unclear whether the method could work effectively in natural ecosystems. This first example aims to enable biodegradation under varied conditions; others aim to utilize a change in specific environmental factors to trigger biodegradation. Kikkawa et al. developed a method to initiate biodegradation only upon exposure to light.^[^ ^257^ ^]^ They developed a photo‐triggered enzymatic degradation mechanism for biodegradable plastics, by applying a layer of a light‐responsive Azo compound onto a thin film of poly(L‐lactic acid) (PLLA). When exposed to UV light (365 nm), the Azo compound melts, enabling the enzyme (proteinase K) to reach the underlying PLLA and facilitating controlled degradation. This approach offers a potential solution to time the start of biodegradation processes. However, further research is required, as the degradation trigger occurs only with a specific wavelength of UV light. Consequently, it is necessary to test the performance of such film under conditions of direct and indirect sunlight to determine whether it can be effectively triggered or if a certain amount of sunlight accumulation is required.

Although first steps for developing more intelligent biodegradable materials have been demonstrated, the development of more capable materials that combine durability with controlled biodegradation that can occur across diverse ecosystems is highly needed. Integrating multiple material solutions into a functional composite is potentially an effective way to achieve the complex behaviors necessary for ESR materials.

### 8.2 Technological Challenges

To increase the capabilities of ESRs for enabling remote‐controlled or autonomous operation, it is necessary to integrate actuation, sensing, computation, communication, and energy storage or harvesting into the robotic platform, thereby allowing for ESRs with increased complexity.^[^ ^249^ ^]^ Progress has been made on individual components, but these components have not reached the level required to enable the reliable integration of multiple components into ESRs. Due to this bottleneck, research has focused on the development of ESRs with low complexity, using functional materials.^[^ ^225^, ^226^ ^]^ In such devices, energy is stored as elastic energy or harnessed from the environment itself, for example, through utilizing humidity changes,^[^ ^222^, ^224^ ^]^ temperature changes,^[^ ^5^, ^225^ ^]^ or wind.^[^ ^209^, ^211^ ^]^ Similarly, their control is outsourced to the environment as well, as functional materials react to environmental stimuli. Currently, sensing and communication of data are mostly achieved by remotely recording of the sensor's current state through a passing UAV or other agent, as ESRs with low complexity lack the capability of data storage and active data transmission to a receiver.

ESRs that locomote for longer distances (a few body lengths or more) on land or in water require controllable actuators and untethered operation. So far, controlled actuation using biodegradable soft actuators has only been achieved in tethered configurations, wherein the actuators are driven by non‐degradable power supplies and control.^[^ ^232^, ^236^, ^240^ ^]^ Although tethered actuators allow for biodegradable end effectors, they limit the application of ESRs or robots in general in natural ecosystems.^[^ ^258^ ^]^ Increased efforts toward untethered electrically or chemically driven actuators are needed to achieve this goal. While the first electrically driven biodegradable actuators have been demonstrated,^[^ ^229^ ^]^ most polymer‐based biodegradable actuators are pneumatically driven,^[^ ^228^, ^230^, ^231^, ^234^ ^]^ making untethered operation more difficult, due to bulky pumps and pressure controllers needed for pneumatic actuation.^[^ ^259^ ^]^ Creating energy‐autonomous ESRs with greater complexity requires the ability to store energy and harvest it, especially for prolonged use in the field.^[^ ^260^ ^]^

A long‐term challenge will be to produce sophisticated computation and communication systems that do not generate electronic waste and pollute the environment. Currently, such technology is mostly based on silicon electronics due to their miniaturization and scaled‐up production. If high performance of electronic systems is important, it is unlikely that electronics can be realized with biodegradable alternatives. Hybrid approaches, in which electronic units are retrievable, might present a practical compromise.

### 8.3 Environmental Challenges

When it comes to deploying ESRs in natural ecosystems, the main challenge is enabling ecosystem‐specific biodegradability. While biodegradable materials have been mostly assessed for disposal in waste treatment facilities, field tests in terrestrial ecosystems are lacking.^[^ ^48^ ^]^ Similarly, more research on the biodegradability of materials in aquatic ecosystems is needed. An additional challenge arises from the source of the materials used. Many biodegradable, biohybrid, and bioinspired structures contain constituents or components that come from various organisms.^[^ ^261^ ^]^ It is essential to prevent the introduction of invasive species to different ecosystems, as they may be harmful even if they biodegrade into the environment.^[^ ^262^ ^]^ A possible solution to these challenges is to harvest resources for material preparation from the same ecosystem to which the ESR is deployed. For example, proteins or polysaccharides could be sourced from aquatic flora and fauna, which has shown promise in recent years for developing new aquatic ESRs.^[^ ^263^ ^]^

Another challenge is the constantly changing environment to which these ESRs are intended to be deployed. Even now, we see that ecosystems are adapting to climate change in different ways.^[^ ^264^ ^]^ Specifically, when deserts transform into grasslands for extended periods, known as desert greening, the landscape and environment are completely altered, necessitating an ecosystem‐specific design that can adapt to the changing climate.^[^ ^265^, ^266^ ^]^ Conditions within an ecosystem become more extreme due to climate change, such as ocean acidification causing changes in calcium carbonate levels in coral reefs,^[^ ^267^ ^]^ broadening their range. While some changes might benefit the biodegradability of materials in specific ecosystems, they may also increase the requirements for the performance and durability of materials (i.e., the increase of natural disasters).^[^ ^268^ ^]^ Ultimately, changing ecosystems will require technology versatile enough to accommodate these transformations. Better monitoring and prediction of developments in ecosystems will inform the development of ESRs and assist in maintaining our planet's health.

### 8.4 Testing ESRs in the Field

Once the materials and components are more robustly fabricated and the autonomy of untethered ESRs has sufficiently progressed, testing new technology in the field becomes an additional challenge. Finding testing sites that allow evaluation and iterative development of ESRs in given environments is a crucial part of the design process. A solution to this problem in ecosystem‐centered design would require a controlled ecosphere or a controlled artificial ecosystem to test the deployment of ESRs and their biodegradability. Zoos, aquariums, and botanical gardens could simulate such ecosystems because they often mimic the conditions of specific ecosystems.^[^ ^200^, ^269^ ^]^ Researchers developing ESR technology have already used this approach to test technologies in controlled ecospheres.^[^ ^231^, ^270^ ^]^ An advantage of these controlled environments is that they provide a controlled system where testing can mitigate adverse side effects, such as the introduction of invasive species (including plants or fungi) that are part of the design process.

### 8.5 Future Research Directions for ESRs

To address the material challenges for developing ESRs, it is necessary to develop materials that exhibit enhanced performance tailored to specific ecosystems. These materials could also offer possibilities for controlled biodegradation. Implementing biodegradation triggers is a promising avenue of research to achieve a balance of stability and biodegradation. Before this approach is implemented into an ESR, researchers must have a thorough understanding of the environmental factors of an ecosystem and how they change over the operation time of a robot. When researchers have knowledge of the environmental factors, they can initiate degradation in response to the conditions encountered in the deployed environment. Once triggered, effective degradation needs to be facilitated. Incorporating enzymes to aid biodegradation, as presented by Huang et al.,^[^ ^256^ ^]^ represents a promising avenue for the future to achieve controlled biodegradation in diverse ecosystems. An ecosystem‐centered approach that incorporates environmental cues to inform operation and biodegradation behavior could enable more capable materials and robots. A potential example of this strategy would be providing embodied intelligence in a robotic system through responses to multiple stimuli. This embodiment could be realized through actuation, triggered biodegradation, or even self‐healing. An example research avenue of embodied intelligence with biodegradability could be studying how hydrogels may achieve reversible actuation in response to humidity; yet initiate depolymerization upon crossing a defined pH or temperature threshold. Integrating actuation, sensing, responding, and biodegradation is feasible if careful measures are implemented to prevent conflicts between functionalities under the same environmental conditions.

When environmental cues are insufficient for complex behavior more conventional approaches (i.e., using digital control) should be employed. We propose these ESRs could be hybrid incorporating biodegradable materials with non‐biodegradable electronics as a first step in realizing complex ESRs. In such a solution, researchers should work to make most of the robot's mass and volume ecoresorbable. The computation and communication unit should be integrated in a way that minimizes its effect on the ecosystem or allows researchers to retrieve the non ecoresorbable components more easily. For example, a control unit could float to the water surface after separating from the main body, where it can be collected by researchers. Additionally, combining digital computation with physical forms of computation, such as embodied mechanical intelligence or material intelligence, could help achieve more complex behaviors. Utilizing these intelligent designs should simultaneously help reduce cost for digital computation, allowing for simpler or smaller electronic components.

We believe making newly developed materials or robots accessible to a variety of research groups and research fields is necessary to accelerate the development of ESRs. The development of ESRs is a highly interdisciplinary endeavor that can only be successfully tackled if engineers, materials scientists, environmental engineers, biologists, and roboticists work together. Material fabrication methods recently have become more accessible through tools like mold casting,^[^ ^271^ ^]^ 3D printing,^[^ ^272^ ^]^ or direct ink writing,^[^ ^273^ ^]^ allowing easier interdisciplinary endeavors. These fabrication techniques allow for rapid prototyping helping create prototyped solutions for researchers to exchange and iterate through ideas to non‐specialized research groups.^[^ ^274^ ^]^ This aspect is particularly important for shortening the development time of ESRs until more ecoresorbable materials and components are commercially available.

## 9 Conclusion

The world's immense biodiversity, hosting numerous ecosystems that provide a thriving environment for flora and fauna, requires our support to maintain this diversity and our planet's health. Sustainability robots, operating in those ecosystems, can play a crucial role in this endeavor. However, if not designed appropriately, such technological aids, particularly when deployed at scale, can disrupt, pollute, or damage ecosystems. At the end of their life cycle, sustainability robots should either be retrieved or biodegraded at their deployment site. The preferred strategy depends on the type and location of the ecosystem, as well as the environmental conditions that facilitate biodegradation. In this review, we categorized ecosystems to guide the development process of sustainability robots, indicating which ecosystems are more likely to benefit from retrieval or biodegradation at the robots' end‐of‐life. We introduced the term ESRs for robots that can safely disintegrate in an ecosystem, highlighting current developments in this area. Current ESRs are mostly based on functional materials that can react to environmental changes, but more research is needed to increase the complexity of ESRs. Achieving ESRs capable of tackling future challenges will necessitate a careful co‐design of materials, robot components, functionality, and biodegradability. The properties and conditions of the deployment site (e.g., UV exposure, soil type, pH, and temperature) must be included in the development process of ESRs to ensure biodegradation in a specific ecosystem. An ecosystem‐centered design approach, which takes into account a biodegradable material loop, robotic design loop, and ecoresorbable robotics loop, specific to the ecosystem of interest, will be the most effective and least harmful way to create future ESRs.

## Conflict of Interest

The authors declare no conflict of interest.

## Supporting information

Supporting Information
