# Supplementary material for: Ecosystem‐Centered Robot Design: Toward Ecoresorbable Sustainability Robots (ESRs)
Source: Adv Sci (Weinh). 2025 Dec 19;13(15):e09194. doi: 10.1002/advs.202509194 (PMC13042858; doi:10.1002/advs.202509194)
Supplement: Supplementary file 1 — Supporting Information [file ADVS-13-e09194-s001.pdf]

Supporting Information - Ecosystem-Centered Robot Design: Toward Ecoresorbable Sustainability Robots (ESRs)

Tülin Yılmaz Nayır[+,1,2], Yuan Fang[+,1], Consuelo Contreras[+,1], Andrew K. Schulz[3,*], and Florian Hartmann[1,*]

[1]Biomimetic Materials and Machines Group, Max Planck Institute for Intelligent Systems, Stuttgart, 70569 Germany

[2]Environmental Engineering Department, Faculty of Engineering, Gebze Technical University, Kocaeli, 41400 Turkey

[3]Haptic Intelligence Department, Max Planck Institute for Intelligent Systems, Stuttgart, 70569 Germany

+ these authors contributed equally

* correspondence to: aschulz@is.mpg.de, hartmann@is.mpg.de

This PDF file includes:

Supporting Information
Tables S1-S7

Supporting Information

Detailed Classification of Ecosystems Biotic and Abiotic Factors

Every ecosystem presents unique challenges and advantages for biodegradation, making it essential to classify them based on how environmental conditions facilitate biodegradation. For instance, warm and humid ecosystems enhance microbial activity, while extreme conditions such as dry deserts or cold polar regions may slow down or prevent biodegradation. By analyzing these ecosystems, researchers can better understand what design and material choices must be considered for truly ecoresorbable robotic systems.

To rank individual ecosystems, we reviewed each ecosystem and their environmental conditions, organizing them from least favorable to most beneficial for natural material decomposition. We took a snapshot of each ecosystem, typically with data from the period 2000 to 2015 and in some cases more recent data. This snapshot of time allows future researchers to compare and contrast these ecosystem environmental conditions and can note if/when conditions fluctuate because of climate change.

Terrestrial Ecosystems

This ecosystem class encompasses twelve natural terrestrial ecosystems, eleven of which are described and grouped by Cheng et al. [1], including polar, four types of grasslands, three types of deserts, three types of forests, and caves. We describe each of these ecosystems in more detail in the following subsections.

Polar:

Polar ecosystems refer to the latitudinal regions where the mean annual biotemperature is between 0 °C and 1.5 °C, such as the High Arctic (areas above approximately 70 °N) and Antarctica [2]. Additionally, altitudinal belts are also determined by biotemperature, resulting in an equivalence between latitudinal regions and altitudinal belts. For example, the nival belt above 4750 m is also classified as a polar ecosystem, with the high Tibetan plateau where the elevation exceeds 5000 m as one of its representatives [3].

Polar ecosystems are characterized by a persistently frigid climate, and they are the most unfavorable ecosystems for biodegradation. An example is demonstrated in the Canadian High Arctic, where the minimum temperature can drop to −27.8 °C and the maximum temperature remains only 3.5 °C [4]. The cold climate of polar ecosystems also leads to a low annual precipitation of less than 200 mm [5], as the air is unable to retain water vapor to form clouds. The UV radiance in polar ecosystems is very low. At the South Pole, the UV index is typically less than 4 [6], primarily due to the low solar elevation, which increases the optical path length of solar radiation. As a result, the UV radiation is mostly absorbed and scattered before reaching the Earth's surface.

The mechanical force in polar ecosystems is the strongest among all ecosystems, with an annual average wind speed of up to $40\,\mathrm{m\,s^{-1}}$ [7]. Owing to the low precipitation and strong wind, the soil in polar ecosystems is dry and silty. The dry soil, particularly permafrost, acts as a barrier, trapping salt in the soil surface and resulting in high soil salinity [8]. The dry soil lacks essential nutrients such as carbon, nitrogen, and phosphorus [9, 10], which are necessary food for bacteria. The most available microorganisms in polar ecosystems are metabolically adaptable aerobes, including Firmicutes, Acidobacteria, and Actinobacteria [11, 9]. The pH value of the dry saline soil is around 7.9-9 [9].

Grasslands

Grassland ecosystems are characterized by grass-dominated vegetation, including grasses, sedges, and herbaceous plants as the primary types. Trees are either sparse or absent, typically found only in areas with more moisture or fewer disturbances, such as fires or grazing. Due to the open nature of grasslands, these ecosystems receive high levels of radiation, as they are not shaded by dense forest canopies, except in the tundra and alpine steppe, where the UV radiance is very low. These ecosystems also experience significant seasonal variations. For instance, tundras, alpine steppes, and temperate humid grasslands have cold winters and warm summers. In contrast, savannahs undergo intense wet and dry seasonal cycles, while steppe ecosystems can vary from temperate to cold, often with considerable temperature fluctuations.

Tundras and Alpine Steppes:

The treeless tundra ecosystems, featured with low summer temperatures of around 10 °C, consist of arctic tundra and alpine steppes [12]. Since there is no definite boundary between the two sub-ecosystems, we will focus on the tundras in the Arctic Circle. In Arctic tundras, the annual precipitation is about 400 mm [13], and the UV radiance is very low, with a UV index of 1.5 [14]. The wind in tundra ecosystems is very strong, which is beneficial for mechanical erosion of polymers [12]. The soil is composed of 57 % silt and clay with abundant carbon and nitrogen [13] but low available phosphorus [15, 16]. Furthermore, the soil is very acidic with a low pH value of 4.58, which is conducive to chemical erosion of biodegradable materials [13]. Due to the fertile soil, the microorganisms in tundra ecosystems are abundant. For example, the bacterial richness and relative abundance in Arctic tundra are similar to those in other ecosystems such as tropical and temperate forests [17].

Steppes:

The second ecosystem in this category are steppes, a semi-arid type of grassland. Steppes have an annual mean temperature ranging from 0 to 3 °C and receive between 250 to 350 mm of rainfall yearly [18]. Wind is the primary mechanical force aiding in the abiotic degradation of materials in steppes, especially prevalent in the spring and winter seasons, with an annual average wind speed of $4.5\,\mathrm{m\,s^{-1}}$; there are also days when the wind speed exceeds $17\,\mathrm{m\,s^{-1}}$. The soil found in this ecosystem is typically chestnut soil, characterized by a high sand content, which results in low to moderate fertility [19]. The soil in steppes is also slightly alkaline, with a pH value between 7.0 and 8.1 [20]. In steppe ecosystems trees are scarce, therefore the surface soil receives ample sunlight, which allows bacteria to breathe and thrive [21].

Temperate Humid Grassland:

The next ecosystem in this category is temperate humid grasslands. Owed to the synergistic effect of the warm climate and high moisture, temperate humid grasslands have a higher annual average rainfall of 494 mm [22]. Temperate humid grasslands are similar to steppes as wind remains the dominant mechanical force in this ecosystem, with the annual average wind speeds ranging from 3.8 to $6.1\,\mathrm{m\,s^{-1}}$. In temperate humid grasslands, there is a considerable amount of UV radiation as a result of more than 2 400 hours of sunlight annually [23]. Moreover, the soil is also rich in essential nutrients such as nitrogen, carbon, and phosphorus [24, 25], contributing to the activity and diversity of microorganisms, with a pH value of 6.4 [25].

Savannahs:

The final grasslands-type ecosystem is savannahs, which are ecosystems that can be found in its majority near the equator. Savannahs are notably warmer than other grassland-type ecosystems, with a mean annual temperature ranging from 25 to 37 °C [26]. Savannah ecosystems show a wet-dry season, as they

have an annual rainfall of 1100 mm, which occurs mainly during a short rainy period. Due to this reason and the high UV index, which usually exceeds 10 [27], these ecosystems present long, dry periods. These long, dry periods foster a climate that gives way to a fire regime with a mean fire return interval of 5.8 and 4.05 years [28]. The soil in savannahs has a slightly acidic to neutral pH value of 6.36 to 6.98 [26] and contains a high amount of silt (10 to 69 %), and fractions of clay [28, 29]. Actinobacteria, Chloroflexi, and Firmicutes dominate the bacterial community [28], all of which help in matter degradation through different ecosystem processes.

Deserts

Deserts are polyextreme environments characterized by large temperature fluctuations, extremely low annual precipitation (0 mm to 400 mm), and scarce vegetation. Deserts can be broadly classified into warm deserts, cold deserts, and semi-deserts, with the latter exhibiting higher precipitation (250 mm to 400 mm) and similar temperature characteristics to cold deserts.

Deserts exhibit substantial seasonal and daily temperature variations, with temperature directly affected by solar radiation due to the lack of vegetation. For example, warm deserts can experience extremely high temperatures during the day due to large-area exposure to solar radiation, while heat is quickly lost at night due to low water vapor content in the air.

Deserts' soils are predominantly composed of sand (70 % to 95 %), with low amounts of silt (3 % to 24 %) and clay (0.5 % to 10 %) [30, 31, 13]. The soil pH value of desert ecosystems tends to be slightly alkaline (6.4 to 8.7) due to the presence of alkaline ions, which are not significantly leached by rainwater. Deserts are also deficient in carbon, nitrogen, and phosphorus due to insufficient organic matter input, such as dead wood and decomposing leaves [32, 33, 34, 35, 36, 37]. In the following text, we provide details of the subtypes of desert ecosystems:

Warm Deserts:

The first subtype of desert ecosystems is warm deserts. Warm deserts exhibit the highest temperature among all terrestrial ecosystems, with their temperature reaching up to 50 °C [38]. This high temperature facilitates biodegradation by melting polymers and improving chain mobility. The UV radiance of warm deserts is also high, with a UV index of up to 9 [39]. The wind in warm deserts is strong, and the pH value of the soil is around 7.9 [13]. The annual precipitation in warm deserts is around 30-300 mm [38]. Similar to semi-desert, which are discussed below, most bacteria in warm deserts are aerobic, but some anaerobic bacteria, such as Bacteroidetes, are also present [13, 40].

Cold Deserts:

Cold deserts are characterized by low monthly average temperatures, which range from (-6 °C to 38 °C) [41]. The Atacama Desert, a typical cold desert, experiences extremely low annual precipitation (<2 mm) [41]. This low annual precipitation causes evaporation to exceed precipitation. The water vapor content is too low to prevent radiative cooling of the land surface, which leads to significant diurnal temperature variations of around 42.6 °C [42]. The high levels of surface UV irradiance in the Atacama Desert, with a UV index of up to 21 in January of each year. This high UV index can facilitate the depolymerization of polymers [43]. The annual average wind speed is around 2.4 $m\,s^{-1}$ [42], and the pH value of cold deserts ranges from 6.4 to 8.4 [32]. Despite the harsh conditions, cold deserts are home to a rich diversity of microorganisms, including many aerobic bacteria like Proteobacteria [30] and a few anaerobic bacteria [44].

Semi-deserts:

The final subtype of desert ecosystems is semi-deserts. Semi-deserts exhibit similar temperature characteristics to cold deserts, with monthly average temperatures ranging from $-8.7\,°C$ to $22.4\,°C$ [45]. Semi-deserts also receive high precipitation values per year ranging from (250 mm to 400 mm) [33]. The UV radiance is lower in semi-deserts than in cold deserts, with the highest UV index of around 6 annually. The annual average wind speed is $1.9\,m\,s^{-1}$ to $2.6\,m\,s^{-1}$ [46], and the soil in semi-deserts is more alkaline, with a pH value around 8.7 [33]. This high pH value in semi-deserts is beneficial for the hydrolysis of biodegradable materials like poly(lactic-co-glycolic acid) (PLGA). Most bacteria in semi-deserts are aerobic, but some anaerobic bacteria, such as Desulfobacterota, are also present [47, 48].

Forests

Forest ecosystems provide ideal conditions for biodegradation due to their unique vegetation structure and humid microclimate. These ecosystems are stratified, with layered canopies consisting of emergent, canopy, understory, and ground layers. The multi-tiered forest structure absorbs and reflects more than 99 % of sunlight [49], with the upper layers capturing most UV radiation and the lower layers absorbing light that passes through. A humid microclimate is found inside these forest ecosystems, characterized by lower annual average wind speed, higher humidity, and moderate temperature fluctuations. Their soils are acidic, with pH values ranging between 4.69 and 5.2. Additionally, forests exhibit high biodiversity compared to other natural ecosystems, recycling nutrients through decomposition and maintaining soil fertility [50, 51, 52]. There are several subtypes of forests with specific environmental factors, which we describe in more detail below:

Tropical Forests:

Tropical forests, primarily located along the equator, receive intense and consistent solar radiation, resulting in a persistently high UV index throughout the year. In winter, the UV index of tropical forests reaches 8, and in summer, it rises up to 16 [53]. Under optimal climatic conditions, the soil in tropical forests is fertile and mainly composed of clay. For example, in the Lappwald forest, the soil is derived from ancient rock, specifically clay formations dating back to the Triassic and Jurassic periods. This ancient clay would typically result in nutrient-rich soil; however, due to the constant rain (more than 2600 mm annually) and surface water flux. Instead, those rocks are now covered by a younger river-deposited soil layer made of sandy clay loam [54, 55].

The fertile soil in tropics forests fosters a large amount of microbial activities and biodiversity. As a result, there are abundant bacterial phyla, including Proteobacteria, Planctomycetes, Nitrospirae, Verrucomicrobia, Bacteroidetes, Chloroflexi, and Actinobacteria all are found in tropical forests. These diverse bacteria are involved in multiple ecological processes, such as the carbon cycle, nitrite oxidation, photosynthesis, and organic matter degradation and decomposition, further promoting soil nutrient enrichment [56].

Temperate Forests:

Temperate forests are characterized by high annual precipitation, which is demonstrated by some temperate forests receiving up to 7000 mm of rainfall yearly [50]. Temperate forests are located in mid-latitudes where the UV radiation is low showing a lower UV index varying from 0 to 7. In temperate forests, the components of the soil are complex and can be classified as sandy loam, loam, or silt loam. For example, an evergreen temperate forest in Southern Chile has soil composed of andesitic and basaltic tuff, scoria, and sand of different particle sizes [50], which is typical of volcanic soil. Bacterial communit-

ies in this ecosystem include Rhizobiales and Acidobacteria [57], with the former responsible for fixing atmospheric nitrogen (N_2) and the latter contributing to the decomposition of organic matter.

Subtropical Forests:

Subtropical forests are positioned latitudinally between the two other types of forests between tropical and temperate. Subtropical forests are typically located beyond the tropics, and have two seasonal climates: humid and warm in summer and mild in winter. Subtropical forests receive an average annual rainfall of 1420 mm, with a mean temperature of 23.5 °C. The soil in subtropical forests is primarily yellow and red earth, mainly consisting of medium loam and heavy loam, and has a low to moderate fertility [58]. Despite these conditions, subtropical forests supports a diverse range of microbes, including 53 phyla, 190 classes, and 359 orders of prokaryotes, as well as seven phyla, 35 classes, and 120 orders of fungi [51].

Caves:

The final terrestrial ecosystem we describe is caves. Caves are characterized by total darkness or low light level. These features combined with high humidity, and a sharp reduction in oxygen levels from the entrance to deeper, narrow passages create cave ecosystems [59]. Medina et al. conducted a review of 12 caves located in different climatic zones, from tropical to cold continental climates, and found that their typical temperatures range from 2 °C to 26.7 °C [60].

Rockfalls and small amounts of water flow contribute to the physical movement of materials. These in-cave flow systems transport both organic and inorganic matter from the surface to the cave sediment [61]. Water availability inside caves is measured somewhat indirectly. As caves are often subterranean the only way to access water is by water dripping or flowing from the surface, and the drip-water ratio depends substantially on the season. In rainy months, the dripping water ratio could reach 60 drips per minute, which is increased hundredfold to 5 000 drips per minute during storms [62]. Caves can contain varied sediment deposits, including minerals, organic matter, and chemical deposits such as phosphates and nitrates [61]. Although caves can contain a diverse array of sediment deposits the nutrient level is typically low [63].

Despite the low nutrient levels, a high degree of biodiversity exists within the Bacteria and Archaea domains in caves. The composition of microbial diversity is impacted by the cave's pH value, which varies with the cave's geological composition and mineral content. An example is shown in limestone caves, which often exhibit a pH range between 7 to 8. This is different when compared to volcanic or silica-based caves which exhibit more acidic conditions as the pH value range is lower than limestone caves, ranging between 4 to 6 [63].

Aquatic Ecosystems

Water occupies approximately 71 % of the Earth's surface in various forms, including oceans, seas, rivers, lakes, and underground water networks [64]. Here, we categorize bodies of water first by their salinity (i.e., saline or fresh water) and proceed to further sub-categorize by distinct depths (i.e., water surface or underwater).

Saline Waters

Saline waters have a typical salinity in the range of 34 ppt to 36 ppt, account for approximately 97.5 % of the world's water in the form of seas and oceans [64]. Sea surfaces and deep sea environments, both part of saline waters, have different physical conditions and nutrient sources. The surface waters

receive more sunlight and oxygen than regions underwater, leading to differences in their temperature and oxygen characteristics. While 5 % to 10 % of solar radiation is reflected at the water surface, light reaches through the top layers of water. Due to the absorption spectrum of water, UV light in the range of 320 nm to 400 nm exhibits the highest penetration, where the radiance attenuates to 10 % of its original intensity at a depth of 38 m [65]. Sunlight on the water surface is critical for biodegradability as it enables photosynthesis, which generates organic matter and supports biologic activity. In contrast, the dark and oxygen-poor deep sea depends on sinking inorganic particles, dead cells, and other detritus from upper layers [66].

Sea Surfaces:

Sea surfaces, which for our classification included both nearshore and offshore regions, plays a key role in the marine ecosystem. As the interface between the deep seas and the atmosphere, sea surfaces fulfill multiple essential functions. These functions include solar energy absorption, gas exchange, and nutrient redistribution all of which are critical to our Earth's biome. The temperature of sea surfaces varies depending on climatic conditions, but it is typically reported to range from 10 °C to 20 °C [64]. Sea surfaces are rich in oxygen, with wind and currents acting as mechanical forces that help aerate the water [64]. The pH of sea surfaces is generally around 8 [67]. While nutrient amounts are low and stable in the open seas, nearshore waters show variations due to exposure to domestic and industrial wastewater [64]. Microbial life, including bacteria, algae, diatoms, protozoans, and fungi, constitutes the diverse population of microorganisms in sea surfaces [68].

Deep Seas:

The deep sea is defined as beginning at a water depth of 200 m, where sunlight is no longer sufficiently available for photosynthesis [69]. Due to the decreasing light exposure with depth, the temperature in deep seas is lower than 4 °C [66]. The pH value of deep seas is slightly lower than the sea surfaces, typically ranging between 7.5 to 7.8 [67]. Nutrient levels are higher in deep seas than the sea surfaces. This nutrient abundance in the deep seas creates a favorable environment for microbial life [70]. In the oxygen-poor environment of the deep seas the anaerobic bacteria and fungi thrive [67].

Coral Reefs:

Coral reefs are large underwater structures formed by living organisms, primarily coral polyps and algae, and are characterized by their high levels of flora and fauna biodiversity [71]. Coral reefs generally thrive in warm waters, but regions such as the Arabian Gulf show extreme temperature ranges between 10 °C to 40 °C [72]. The interaction between coral reefs and surrounding water is governed by water flow, tides, and mesoscale currents and waves, as well as the small turbulences [73]. Oxygen is an important parameter for reef functioning and health. Oxygen is produced by corals, algae, and other photosynthetic organisms and consumed by nearly all reef species. At the reef scale, dissolved oxygen concentrations typically range from 3.4 $mg\,L^{-1}$ to 13.6 $mg\,L^{-1}$ [74]. Corals prefer alkaline waters with a pH of 7.9 to 8.3, which are typical values for the ocean [75] and likewise coral reefs. Coral reefs are typically found in nutrient-poor environments [76]. However, microbial diversity, including bacteria and archaea, plays an important role in nutrition and disease resistance of corals [71].

Fresh Water

Fresh water is mainly stored in polar and groundwater ecosystems. Only about 0.25 % of fresh water is held in rivers and lakes [64]. Although small in quantity, these ecosystems are essential for both humans and wildlife to survive.

Groundwater Networks:

Groundwater refers to the water located beneath the Earth's surface, formed by infiltration of precipitation that fills pores and spaces in soil and rock. This groundwater forms networks that create the groundwater network ecosystems. The temperature of groundwater networks can vary substantially (0.43 °C to 31.8 °C) across countries [77]. As the distance from sea surfaces or terrestrial habitats increases, groundwater networks become increasingly depleted in oxygen and nutrients [78]. Compared to saline waters, where dissolved solids primarily consist of sodium and chloride, groundwater networks often contain additional magnesium, calcium, and sulfate. These additional minerals lead to a higher salinity compared to other freshwater systems, ranging from 1000 mg L^{-1} to 10 000 mg L^{-1} [79]. The pH value of groundwater networks is often neutral, but can vary regionally. For example, measurements of a groundwater network in southwestern Germany resulted in a pH range from 6.5 to 7.5 [80]. A neutral pH value is beneficial for microorganisms, which exhibit an abundance of Bacteria, Heterotrophic Flagellata, Amoebae, Ciliata, and Heliozoan [81].

Rivers:

Rivers are very dynamic ecosystems with large variability of conditions depending on: location (even within the same river), variations in solar radiation, and water sources. There are many subtypes of rivers that have distinct climate ranges. Sub-polar, polar, and high plateau rivers are cold (average temperature (T_{mean}): < 8 °C), subtropical and alpine rivers are of intermediate temperatures (T_{mean}: 5 °C to 26 °C), and tropical rivers are warm (T_{mean}: > 25 °C) [82]. Water currents are the primary mechanical force in rivers and are responsible for sediment transport and abrasion of material [83]. Rivers are very low salinity ecosystems (global mean 120 mg L^{-1}) and are typically well-aerated through the continuous water flow [83]. While the pH value for river ecosystems can vary with location, the Danube river's pH was measured between 6.2 to 9 [84]. Nutrient levels in rivers can change depending on the season and location. For example, in spring, due to the snow-melt and subsequent runoff into the rivers organic content in the river might increase. Moreover, rivers that close to agricultural fields might have higher availability of nutrients due to exposure to fertilizers and sewage. In the absence of waste streams, lower nutrient levels are expected. Microbial life in rivers is dominated by bacteria affiliated with the phyla proteobacteria (particularly betaproteobacteria), actinobacteria, bacteroidetes, cyanobacteria, and verrucomicrobia [85].

Lakes (surface):

For saline ecosystems we split the large bodies of water into two subsystems, including the surfaces and the deeper oceans. Similarly, we divide lakes into two distinct zones: the surface and underwater. The lake's surface layer is in direct contact with the atmosphere, where heat transfer and atmospheric gas exchange occur. The temperature of lake surfaces changes with the seasons, particularly between winter and summer, ranging from 3 °C to 25 °C [86]. In summer, lake surfaces warm up, forming a warmer upper layer called the epilimnion, while the cooler layer beneath is known as the hypolimnion. During winter, lake surfaces are often covered by ice, preventing wind-driven mixing. As a result, the deeper layers beneath the lake surface remain relatively warmer and more stable.

In a well-aerated lake, oxygen concentration of lake surfaces is around 12 mg L^{-1} to 13 mg L^{-1} (at 4 °C and 100 % saturation) [83]. The typical pH value for lake surfaces ranges from 6 to 9 [87], and salinity is lower than 1 ppt [64]. Carbon is the most abundant nutrient in lake surfaces and is also the most common element in algal tissue. Nitrogen and phosphorus are less available, with nitrogen typically present in higher concentrations than phosphorus [88]. Elevated phosphorus levels, often due to human waste, can trigger algal blossoms, which may limit light penetration into the lake. Lake surfaces are primarily inhabited by colonial algae or cyanobacteria, heterotrophic bacteria, and protists [88].

Lakes (underwater):

Beneath the sunlit surface of lakes, the layer underwater is darker, colder, and accompanied by sediment deposits, which we describe as the underwater layers of lakes. The temperature in the underwater layers of lakes is more stable than lake surfaces, despite seasonal thermal fluctuations, and typically ranges between 5 °C and 10 °C [86]. Stratification within the lake leads to underwater layers of the lake having distinct microbial processes due to the variations in oxygen, light, and nutrient concentrations between the surfaces and lake bottoms. While production via photosynthesis occurs at the lighted epilimnion layer, decomposition occurs in the underwater layers. In this bottom water region, also known as the profundal zone, bacteria and fungi dominate the decomposition process [88]. There is constant nutrient exchange between lake layers. Detritus from the epilimnion sinks to the profundal zone where nutrients are released. Additionally, aquatic animals migrate daily between lake surfaces and the underwater layers. These migrations lead to feeding and defecating, thereby enriching the bottom sediment with nutrients [88].

Artificial Ecosystems

Artificial ecosystems are natural ecosystems that have been augmented by humans to fulfill human-centric social, economic, or environmental needs. Unlike natural ecosystems, which change through ecological succession (including both ecological and anthropogenic factors) [89], artificial ecosystems are purpose-built to fulfill human-centered needs. These human-centered needs include, but are not limited to food production, urban development, and/or climate resilience. Artificial ecosystems have some or all of their environmental factors augmented and controlled to provide benefits for their specific purposes. In this review, we will not cover urban ecosystems directly, as they are one of our reference points to assess accessibility of ecosystems, and, additionally, end-of-life scenarios are typically outsourced to other (artificial) ecosystems, such as landfills or waste treatment facilities [90]. For the artificial ecosystems we chose specific sites as artificial ecosystems can be created in almost all of the natural ecosystems above (e.g., aquaculture, cactus farming, grassland farming, etc.). Specifically, we chose a Nigerian farmland as an example of the agricultural fields ecosystem and an open German lignite mine as an example of mines ecosystem.

Mines:

Mines are systems of tunnels and pits to extract valuable minerals from the earth's surface. Mines are generally classified into two types: open-pit (surface) mines and underground mines. Mines differ significantly in their physical and chemical conditions depending on a range of factors. Open-pit mines typically have some natural light availability, exposure to oxygen, and water presence due to precipitation. In contrast, underground mines are characterized by complete darkness (if not illuminated artificially), minimal nutrient availability, and are mostly dry. In some cases, oxygen availability is limited due to the absence of artificial or natural ventilation. Both open-pit and underground mines experience mechanical stresses from rock pressure, compression, and abrasion. These stresses contribute to the erosion of surrounding materials and influence microbial processes within the mine environment. For example, an open German lignite mine exhibits temperatures between −12.5 °C and −2.4 °C at 2-m height, a mean annual precipitation between 450 mm and 600 mm, and Arenic and Sydtric Regosols soil type conformed mainly by sand. Due to the activities in the native German region, several artificial mining lakes exist. These mines contain water characterized by nutrient-poor conditions and very high acidity, as their pH values range from 2 to 4 [91]. As a part of mining processes, acid mine drainage (AMD) is a well-known acid runoff process for metal extraction. Acid streams are present in pH 1 to 3.5 in AMD sites [92], which coincides with our example. Mendez-Garcia et al. have reviewed the biodiversity and microbiology of AMD systems, showing microorganisms belonging primarily to the

domains Bacteria, Archaea, and, to a lesser extent, Eukarya (predominantly fungi and algae) [92].

Agricultural Fields:

Agricultural fields are the most controlled ecosystem we evaluated, as crops are cultivated for human food production, livestock raising, or other means. This meaning they need to be very efficient in production. In general, the climate conditions are similar, as crops usually require the same conditions. However, this does not mean that other soil or climate conditions are changing, as some of them depend on where they are located. For example, in an urban agricultural field in Nigeria, the soil is sandy loam, with 76.3 %, 16.9 % silt, and 6.8 % clay, with a very high amount of nutrients, a slightly alkaline pH range from 7.42 to 7.77 [93]. In Morocco, another agricultural field showcases the diverse features of these fields. In the case of the Moroccan field it contains a clay-loam type of soil (47.6 % clay, 41 % silt, 11.4 % sand), a mean temperature of 18.1 °C, a yearly rainfall of 391 mm. This Moroccan agricultural field has a slightly alkaline soil pH of 7 to 7.9 [94]. Alternatively, an agricultural field that is operated as research farm in South Africa exhibits a much higher rainfall at around 575 mm yearly, which happens mainly during summer. This South African field exhibits a similar average temperature of 18 °C, and soil with high amounts of clay [95]. These distinct farms showcase how, although agricultural fields are the most controlled, they also have some of the highest ranges for their features.

Table S1: Rubric for evaluation of ecosystem EAS for various ecosystems.

Rating	**Very low 1**	**Low 2**	**Moderate 3**	**High 4**	**Very high 5**
Closeness to human presence (CHP)	The ecosystem has very little to no human contact.	The ecosystem presents low human contact, but some occasional impact.	The ecosystem has moderate human presence or impact.	The ecosystem has high human presence and/or is regularly exploited.	The ecosystem has been completely transformed by humans, as it is a part of human settlements.
Ease to navigate terrain (ENT)	The ecosystem is impossible for humans to traverse without risk of harm, therefore specialist equipment is mandatory.	The ecosystem is extremely difficult for humans to traverse without specialist equipment.	The ecosystem is challenging, but manageable for humans to navigate. Specialized equipment can assist.	The ecosystem is easy for humans to traverse without the need of specialized equipment.	The ecosystem is designed for human access and movements.
Mildness of conditions (MoC)	The ecosystem's conditions presents extreme fluctuations or very harsh conditions.	The ecosystem experiences significant changes in climate within a day, or it remains quite harsh.	The ecosystem presents noticeable variations or stable uncomfortable conditions, but generally survivable.	The ecosystem has mild variations, mostly stable.	The ecosystem is very stable and comfortable for humans.

Table S2: Rubric for evaluation of BES for various ecosystems.

Rating	**Very low 1**	**Low 2**	**Moderate 3**	**High 4**	**Very high 5**
Nutrients amount* C, N, P	The ecosystem presents a low amount of microorganism growing, indicating a low amount of nutrients.	-	-	-	The ecosystem presents a high amount of microorganism growing, indicating a high amount of nutrients.
Microbial abundance* [96, 97]	The ecosystem presents in its majority anaerobic-type microorganism.	-	The ecosystem presents in its majority facultative-type microorganism.	-	The ecosystem presents in its majority aerobic-type microorganism.
Water availability* [98]	The ecosystem has less than 600 mm of average annual rainfall.	The ecosystem has an average annual rainfall between 600 mm to 733 mm.	The ecosystem presents an average annual rainfall between 733 mm to 866 mm.	The ecosystem has an average annual rainfall between 866 mm to 1000 mm.	The ecosystem has an average annual rainfall higher than 1000 mm.
Temperature [99]	The ecosystem presents a mean temperature is lower than 10 °C.	The ecosystem's temperature ranges between 10 °C to 20 °C, or has a mean temperature in that range.	The ecosystem's temperature ranges between 20 °C to 30 °C, or has a mean temperature in that range.	The ecosystem's temperature ranges between 30 °C to 40 °C, or has a mean temperature in that range.	The ecosystem's mean temperature is higher than 40 °C.
UV/Light exposure [100, 101]	The ecosystem's UV index is lower than 5.	The ecosystem's UV index is 5 or 6.	The ecosystem's UV index is 7 or 8.	The ecosystem's UV index is 9 or 10.	The ecosystem's UV index is higher than 10.
Oxygen amount [102]	The ecosystem is low in oxygen.	-	-	-	The ecosystem has abundant access to oxygen.
Mechanical forces [103, 104, 67]	In the ecosystem, there is no presence of mechanical forces.	In the ecosystem, there is some presence of mechanical forces sporadically.	In the ecosystem, there is presence of one constant mechanical force.	In the ecosystem, there is presence of multiple mechanical forces sporadically.	In the ecosystem, there is a constant presence of multiple mechanical forces.
pH value [99]	The pH value in the ecosystem is slightly acidic or basic, bordering on neutral, i.e., it ranges between 6 and 8, excluding both extremes.	If the ecosystem's pH range falls into the previous category, and the next one, it means values are from 5 to 9.	The ecosystem's pH values are between 5 and 6 (weakly acidic) or between 8 and 9 (moderately basic).	If the ecosystem's pH range falls into the previous category, and the next one, it means values are lower than 5 up to 6 or from 8 to higher than 9.	The ecosystem presents highly acidic pH (lower than 5) or strongly basic pH (higher than 9) values.
Soil properties, only for land [105]	The ecosystem presents a larger presence of sand or silt.	-	The ecosystem's soil is a mix of silt, sand, and clay.	-	The ecosystem presents a larger amount of clay.
Salinity, only for aquatic ecosystems [106]	The ecosystem has a high presence of mineral salts, greater than 15 ppt.	-	The ecosystems have a moderate presence of mineral salts, from 15 to 1 ppt.	-	The ecosystem lacks the presence of mineral salts, less than 1.

*Biotic factor

Table S3: Detailed ratings with notes for closeness of human presence (CHP), difficult to navigate terrain (DNT), and mildness of conditions (MoC), which factor into the total ecosystem accessibility score (EAS).

Ecosystems	Comment	CHP	Comment	ENT	Comment	MoC	EAS
Agricultural fields	The ecosystem has been completely created by humans.	5	The ecosystem is designed for human access and movement, with infrastructure and pathways built to support regular human activity.	5	The ecosystem is very stable and comfortable for humans, having been modified to maintain optimal conditions for human presence and activity.	5	12
Temperate forests	The ecosystem has been completely transformed by humans, with consistent proximity to and integration with urban and transportation networks.	5	The ecosystem is easy for humans to traverse without the need for specialized equipment, with trails and open areas facilitating movement.	4	The ecosystem is very stable and comfortable for humans, with only minor daily fluctuations that do not affect habitability.	5	11
Temperate humid grasslands	The ecosystem has high human presence and/or is regularly exploited, with widespread land conversion for agriculture and habitation.	4	The ecosystem is easy for humans to traverse without the need for specialized equipment, with most areas accessible and some cultivated zones.	4	The ecosystem is very stable and comfortable for humans, with consistently moderate temperatures and minimal environmental fluctuations.	5	10
Mines	The ecosystem has been completely transformed by humans, with direct integration into urban, industrial, and settlement systems.	5	The ecosystem is challenging but manageable for humans to navigate, with some areas designed for access, though deep zones pose safety risks; specialized equipment can assist.	3	The ecosystem has mild variations, with a consistently stable climate that remains within comfortable human tolerance levels.	4	9
Savannahs	The ecosystem has moderate human presence, with natural areas coexisting near human settlements.	3	The ecosystem is easy for humans to traverse without the need for specialized equipment, with open, well-defined paths and minimal obstacles.	4	The ecosystem has mild variations, with temperature changes between day and night that are within tolerable limits and do not pose significant risks.	4	8
Tropical forests	The ecosystem has high human presence and/or is regularly exploited, with extensive areas impacted despite large remote zones.	4	The ecosystem is challenging but manageable for humans to navigate, with thick vegetation and high humidity; specialized equipment can assist.	3	The ecosystem presents stable uncomfortable conditions, with persistent heat and humidity posing health risks, though resources are sufficient for survival.	3	7
Rivers	The ecosystem has been completely transformed by humans, with nearly all areas in close proximity to or integrated with human infrastructure.	5	The ecosystem is extremely difficult for humans to traverse without specialist equipment, due to strong currents and hazardous water conditions.	2	The ecosystem presents stable uncomfortable conditions, with localized climate shifts that affect microhabitats but do not disrupt overall stability.	3	7
Steppes	The ecosystem has moderate human presence, with partial land use for agriculture while significant natural areas persist.	3	The ecosystem is easy for humans to traverse without the need for specialized equipment, with flat, open terrain dominating the landscape.	4	The ecosystem presents noticeable variations and stable uncomfortable conditions, with dryness and notable diurnal temperature shifts that affect human comfort.	3	7
Subtropical forests	The ecosystem has moderate human presence, with some remote zones but frequent proximity to human activity.	3	The ecosystem is challenging but manageable for humans to navigate, with dense vegetation, high humidity, and tough terrain; specialized equipment can assist.	3	The ecosystem presents stable uncomfortable conditions, with high heat, humidity, and insect activity, though generally survivable with adaptation.	3	6
Lakes (surface)	The ecosystem has high human presence and/or is regularly exploited, with significant proximity to urban centers, though some remote areas remain.	4	The ecosystem is extremely difficult for humans to traverse without specialist equipment, though it remains reachable via boat.	2	The ecosystem presents stable uncomfortable conditions, with regional climate influences creating localized challenges despite overall stability.	3	6
Coral reefs	The ecosystem has high human presence and/or is regularly exploited, particularly in coastal zones due to urban, tourism, and fishing activities.	4	The ecosystem is extremely difficult for humans to traverse without specialist equipment, requiring diving or boat access with significant challenges.	2	The ecosystem experiences significant changes in climate with stable temperatures, but frequent and unpredictable storms and tidal events that pose risks.	2	5
Groundwater networks	The ecosystem experiences low human contact, though it is exploited indirectly through resource extraction.	2	The ecosystem is impossible for humans to traverse without risk of harm, requiring specialist diving and exploration equipment for safe navigation.	1	The ecosystem is very stable and comfortable for humans, with consistently favorable temperature and humidity levels that support long-term habitation.	5	5
Caves	The ecosystem has very little to no human contact, with only limited exploration in select areas.	1	The ecosystem is impossible for humans to traverse without risk of harm, requiring specialist equipment for climbing, diving, or navigation in dark, unstable environments.	1	The ecosystem is very stable and comfortable for humans, with a uniform climate that lacks seasonal or daily variation.	5	4
Sea surfaces	The ecosystem presents low human contact overall, with only coastal zones experiencing occasional human influence.	2	The ecosystem is extremely difficult for humans to traverse without specialist equipment, though access is possible via boat or aircraft.	2	The ecosystem experiences no significant changes in climate within a day, but remains harsh for humans, with high exposure and risk of sudden storms, increasing danger.	2	3
Warm deserts	The ecosystem presents low human contact, though some areas are impacted by tourism and localized human presence.	2	The ecosystem is challenging but manageable for humans to navigate, with extreme heat posing significant challenges; specialized equipment can assist.	3	The ecosystem's conditions present extreme fluctuations, with dramatic diurnal temperature variations that pose significant physiological stress to humans.	1	3
Semi-deserts	The ecosystem experiences low human contact, with occasional impacts from pastoral activities and small-scale settlements.	2	The ecosystem is challenging but manageable for humans to navigate; specialized equipment can assist in overcoming heat, cold, or sunlight exposure.	3	The ecosystem's conditions present extreme fluctuations, with notable temperature shifts, though less severe than other deserts.	1	3
Cold deserts	The ecosystem has very little to no human contact, primarily due to extreme environmental conditions limiting access.	1	The ecosystem is challenging but manageable for humans to navigate, with generally flat terrain; specialized equipment can assist in certain conditions.	3	The ecosystem's conditions present extreme fluctuations, with intense heat during the day and severe cold at night, creating hazardous thermal extremes.	1	2
Lakes (underwater)	The ecosystem presents low human contact, with only occasional impacts in select areas near human activity, while the majority of the region remains isolated and undisturbed.	2	The ecosystem is impossible for humans to traverse without risk of harm, requiring specialist diving equipment and complex access protocols.	1	The ecosystem remains quite harsh, with extreme cold and oxygen deprivation at depth, making it hazardous for human survival.	2	2
Tundra & alpine steppes	The ecosystem has very little to no human contact, though limited access occurs for scientific or resource-related purposes.	1	The ecosystem is impossible for humans to traverse without risk of harm, requiring specialist equipment due to extreme terrain and lack of infrastructure.	1	The ecosystem experiences significant changes in climate within a day, with persistent cold, strong winds, and unpredictable weather shifts that challenge human adaptability.	2	1
Deep sea	The ecosystem has very little to no human contact, with no significant human presence or infrastructure.	1	The ecosystem is impossible for humans to traverse without risk of harm, requiring specialist equipment due to extreme pressure and hazardous conditions.	1	The ecosystem's conditions present extreme harshness, with consistently freezing temperatures that make survival difficult without specialized protection.	1	0
Polar	The ecosystem has very little to no human contact, with only minimal scientific presence at isolated research stations.	1	The ecosystem is impossible for humans to traverse without risk of harm, requiring specialist equipment due to extreme cold, ice, and absence of infrastructure.	1	The ecosystem's conditions present extreme fluctuations and harshness, with prolonged cold, high winds, and extreme seasonal light cycles that challenge human endurance.	1	0

Table S4: Detailed ratings with notes for the three biotic factors (BF1-BF3) for evaluation of ecosystems' BES.

	Nutrients amount							
	**Carbon amount**	**Nitrogen amount**	**Phosphorus amount**	**BF1**	**Microbial abundance**	**BF2**	**Water availability**	**BF3**
Cold deserts	0.02-0.13%	0.01-2.4%	phosphorus deficiency	1	Bacteria fungi archaea. Lichenised fungi are the most common fungi in cold deserts. The most common bacteria are Proteobacteria which account for 28.1–79.5%.	3	2 mm	1
Semi-deserts	low organic matter content of 1.75 gkg	0.11–0.29 gkg	deficient phosphorus of 0.167 kg/m3	1	Proteobacteria was dominant bacteria phylum.The relative abundance of bacteria at the phylum level in semi-deserts is lower than that in forestland. Ascomycota is the most common fungi in semi-deserts. The archaea mainly belonged to the phyla of Thaumarchaeota. Most bacteria in semi-deserts are aerobic but there're also some anaerobic bacteria like Desulfobacterota.	3	250–400 mm	1
Warm deserts	0.19-1.22%	0.01%	0.0005	1	92% bacteria, 2.75% fungi; most of bacteria in warm deserts are aerobic but still with some anaerobic bacteria like Bacteroidetes.	3	30-300 mm	1
Tundra / alpine steppes	7.02 %	0.00375	low in bioavailable phosphorus, Phosphate concentrations were near or below 1 umol L-1	5	bacterial richness and relative abundance in Arctic tundra is similar to or higher than in other biomes such as boreal, tropical and temperate forests, grassland, desert or prairie. Fungi are dominant in winter than bacteria. Archaea accounted for 0.31% and Acidobacteria were the most abundant phylum within bacteria. In Arctic tundra, there're both aerobic and anaerobic metabolisms	3	400 mm	1
Steppes	18-23 g kg-1	1.5-1.7 g kg-1	666.64 +- 151.0 mg kg-1	5	The culturable bacterial counts in January, April, July and October were 0.13 × 107, 4.09 × 107, 5.33 × 107 and 1.8 × 107 cfu/g, respectively.	5	250 to 350 mm	1
Tem. humid grasslands	2.07 kg m-1	0.1 -0.9 %	10.0 mg kg-1	5	root biomass: 9.8 Mg ha-1	5	494 mm	1
Savannahs	1.29 to 1.9%	0.05-0.11 %	9.28-9.37 mg kg-1	5	microbial biomass: 150-270 mg C kg-1	5	1100 mm	5
Temp. forests	12.3 %	0.56 %	5.9 ppm	5	The temperate forests ... have lichen floras that have the richest and highest biological diversity.	5	7000 mm	5
Subtropical forests	EBL: 46.93 - 51.46 g kg-1<br>DBL: 23.46 - 30.54 g kg-1<br>MCB: 28.25 - 36.81 g kg-1	3.05 +- 0.48 g kg-1	0.35+-0.05 g kg-1	5	We observed 53 phyla, 190 classes, and 359 orders of prokaryotes and seven phyla, 35 classes, and 120 orders of fungi from the 60 soil samples.	5	1420 mm	5
Tropical forests	0.74+-0.06 (OC)%	53.2+-2.4 kg/ha	5.67+-.80 kg/ha	5	High microbial and earthworm activity	5	2651 +- 441 mm	5
Sea surfaces	low	low	low	1	Bacteria, algae, diatom, protozoans, fungi	5		5
Deep sea	higher than surface	higher than surface	higher than surface	5	bacteria, fungi	1		5
Coral reefs	nutrient-poor environments	nutrient-poor environments	nutrient-poor environments	1	bacteria, archea, viruses	1		5
Rivers				1	bacteria (Actinobacteria, Proteobacteria, Bacteroidetes, Verrucomicrobia)	5		5
Lakes (surface)	available (most abundant in different forms)	less (second limiting factor after P)	less (limited for algal growth)	1	Top: colonial algae or cyanobacteria, heterotrofic bacteria, protists (ciliates and flagellates)	5		5
Lakes (underwater)	higher than surface			5	bacteria and fungi	5		5
Groundwater networks		increasing distance from the surface water or terrestrial habitats (up to several hundred metres laterally or vertically), the groundwater becomes more oligotrophic	increasing distance from the surface water or terrestrial habitats (up to several hundred metres laterally or vertically), the groundwater becomes more oligotrophic	1	Bacteria, Heterotrophic Flagellata, Amoebae, Ciliata, Heliozoa	1		5
Caves	oligotrophic ecosystems (less than 2 mg of total organic carbon (TOC) per liter)	oligotrophic ecosystems	oligotrophic ecosystems	1	bacterial phyla, archaeal phyla	3	surface 1100 mm, drip rate 60 per minute	4
Polar	0.0003	0.006-0.014%	phosphate content: 0.36-3.26 µg/g	1	Firmicutes, Acidobacteria, and Actinobacteria were the dominant bacteria. fungal communities contain limited diversity. The most abundant and prevalent microorganisms are metabolically versatile aerobes	5	200 mm	1
Mines (german open mine)	low	low	low	1	Bacteria, Archaea and, to a lesser extent, Eukarya (predominantly fungi and algae)	1	450 to 600 mm	1
Agricultural fields (Nigerian open farmland)	medium to high organic carbon (7.16 – 11.27g/kg)	31.73-213.52 mg/kg moderate to extremely high	high	5		5	1500 mm	5

Table S5: Detailed ratings of six abiotic factors (AF1-AF6) for evaluation of ecosystems BES.

	Temperature	AF1	UV/Light exposure	AF2	Oxygen amount	AF3	Mechanical Forces	AF4	pH value	AF5	Soil prop. or Salinity	AF6
Cold deserts	−6 °C to 38 °C	2	daily UV irradiation doses of ca. 30 J m^2; the UVI in Atacama desert in January is 12 to 21	3		5	annual average wind speed: 2.4 m/s	3	6.4 to 8.4	2	70.2% sand, 24.3% silt, 4.7% clay	1
Semi-deserts	−8.7 °C to 22.4 °C	3	annual sunshine hour of 2674 to 3023 h; The highest UVI in Great basin desert (semi-desert) is 6	2		5	annual average wind speed: 1.9 to 2.6 m/s	3	8.7	3	95.74% sand, 3.72% silt and 0.53% clay	1
Warm deserts	−10 to 50°C	4	high UV radiation. For Mojave Desert, the highest UVI is 7. For Kuwait Desert, UVI doesn't exceed 9.	3	Low	5	Strong wind	3	7.9	1	Sandy loam: 75% sand, 14%silt, 10%clay	1
Tundra / alpine steppes	−20 to 10°C	1	solar diurnal peak is around 200 W/m2 and ground heat flux peak is around 50 W/m2; The UVI is 1.5	1		5	High wind	3	4.58	5	57% silt and clay	3
Steppes	0 to 3°C	1	The surface soil contains more water, enough air, and sunlight for bacteria to breathe and grow.	1		5	Winds predominate in spring and winter, averaging 4.5 ms^{-1}, with 50 to 70 days of 17 ms^{-1}	3	7.0 to 8.1	1	Typical chestnut soil	1
Temp. humid grasslands	5.3°C	1	… greater than 2,400 hours of sunshine annually	2		5	wind speed across the prairies varies between 14 to 22 kmh^{-1}	3	6.4	1	Soil is Orthic Black Chernozem with a clay to clay loam texture	3
Savannahs	25 to 37°C	3	the UVI often exceeds 10 (categorized as 'very high' to 'extreme') and rarely drops below 7	5		5	0.02 0.67 m/s; a lot of fires	4	6.36 to 6.98	1	Appreciable amount of silt (10–69%)	3
Temp. forests	−8.7 °C to 24.8 °C	2	Primary forest floors received a mean of 6.67± 0.29 $molm^{-2}d^{-1}$, and secondary forests $6.47 \pm 0.23 molm^{-2}d^{-1}$	5		5	Strong wind	3	5	3	The material consists of andesitic and basaltic tuff, scoria and sand of different particle sizes	3
Subtropical forests	21.2°C	2	1971 h of sunshine annually	4		5	mean velocity of wind is 1.6 m/s	3	4.69 to 5.56	4	Soils are classified as yellow and red earth, mostly medium loam and heavy loam	3
Tropical forests	23 to 27°C	3	Greater than 99 % of sunlight is absorbed and reflected as the light passes through the forest canopy, resulting in low light intensity and quality in the forest understory …	2		5	Leaves and trunks also exert drag, thereby reducing wind speeds within and below the canopy relative to above the canopy	2	5.2 ± .02	3	Soils of the oak-beech and spruce stand at Lappwald are derived from Triassic sandstone clay and Jurassic clay, and are both covered with a fluvial layer of sandy clay loam	3
Sea surfaces	20 to 30°C	3		5	oxygen rich	5	Wind, Pressure (1 atm at surface increases 1 atm every 10 m), currents	5	8.1 ± 0.2	3	General: 36 ppt tropical: 37 ppt nearshore 36 ppt	1
Deep sea	4 °C	1		1	oxygen poor	1	Pressure is higher than surface	5	7.5 to 7.8	1	34 to 36 ppt	1
Coral reefs	Persian/Arabian Gulf: 10-40 °C	3		1	3.4 to 13.6 mg O_2/L at 27 °C	5	large scales by tides, mesoscale currents, and waves and at the smallest scales by turbulence	5	7.9 to 8.3	2	34 to 36 ppt	1
Rivers	Subtropical and Alpine rivers: TMEAN:5 to 26 °C	2		5	Usually high	5	high flow	3	6.2 to 9.0	2	Global mean: 120 mg/L (around 0.12 ppt)	5
Lakes (surface)	Top: 3 to 25°C;	2		5	12 to 13 mg/L	5	low flow	3	6 to 9	2	1 g/L (around 1 ppt)	5
Lakes (underwater)	Bottom: 5 to 10°C	1		1	absent	1	flow and pressure	4	6 to 9	2	1 g/L (around 1 ppt)	5
Groundwater networks	0.43 to 31.8 °C	2	absent	1	… poorer in oxygen	1	flow, shear stress, sediment transport, pressure changes, and fluctuations	4	6.5 to 7.5	1	TDS major ions, such as sodium (Na), chloride (Cl), magnesium (Mg), calcium (Ca), and sulfate (SO4).	1
Caves	2°C to 26.7°C	2	total darkness or low level of light	1	from entrance to narrow passages, declined to below 18% in a short time	5	Gravity falls such as rockfalls and dripping water, along with water flow	2	2.6 to 3.7	5		1
Polar	−27.8 °C to 3.5 °C	1	Despite the deeper ozone losses and the prevalent clear-sky conditions, the UV index at the South Pole has never been higher than 4	1		5	High annual average wind of 40 m/s	3	7.9 to 9	2	Dry silane soils	1
Mines (german open mine)	−12.5 °C to −2.4 °C at 2-m height	1	open pit mine; light available underground mine; no lights	1	open pit mine; available	5	Rock pressure and compression, Abrasion and erosion	5	2 to 4	5	Arenic and Dystric Regosols	1
Agricultural fields (Nigerian open farmland)	30°C	3	8 to 10	4		5		2	mean pH 7.42 – 7.77	1	Sand: 73.41 to 80.48% Silt: 14.56 to 18.48% Clay: 4.96 to 8.11	3

Table S6: Systematic summary of previously published technologies working towards the realization of ESRs highlighting various aerial and terrestrial environment centered robots and materials.

Technologies	Technology examples	Biodegradable material loop		Robotic design loop			Ecoresorbable robotic loop	
		Primary materials	Biodegradability	Actuators	Controllability	Components	In-situ robotic test	In-situ eco-resorbable test
Drones	Motor-driven edible drones [107]	Puffed rice, non-biodegradable motors and other electronics	Partially biodegradable (no test)	Motors	DC-remotely controlled	actuators, batteries, transmitters, receivers, flight controllers	-	-
	Motor-driven transient drones [108]	Gelatin-cellulose cryogels, carbon-black nanoparticle ink, non-biodegradable electronics	Partially biodegradable (partially degraded after 42 days, norm ISO 20200)	Motors	DC-remotely controlled	actuators, batteries, sensors, transmitters, receivers, flight controllers	-	-
Gliders	Inkjet printed paper gliders [109]	Cellulose paper	Fully biodegradable (fully degraded after 105 days, test in moist soil)	-	EC-autonomous	-	Deployed in artificial ecosystems	-
	Humidity-responsive gliders for pH sensing [110]	Cellulose-gelatin composites, shellac, litmus	Fully biodegradable (70% degraded after 77 days, norm ISO 20200)	Humidity-responsive materials	EC-autonomous	Actuators, sensors	-	-
Sensor holders	Hygroscopically relaxing gripper [111]	Linen, balsa wood, dextrin, gelatin hydrogels	Fully biodegradable (partially degraded in water)	Humidity-responsive materials	EC-autonomous	Actuators	Deployed in forests	-
	Hygroscopically coiling gripper [112]	Cellulose, starch, carbon-loaded shellac ink	Fully biodegradable (no test)	Humidity-responsive materials	EC-autonomous	Actuators	-	-
Wind dispersed sensors	Luminescent fliers for temperature sensing [113]	PLA, fluorescent lanthanide– doped particles	Fully biodegradable (no test)	-	EC-autonomous	Sensors	Deployed in artificial ecosystems	-
	Parachute fliers for multi-sensing [114]	Cellulose acetate	Fully biodegradable (no test)	-	EC-autonomous	Sensors	-	-
	Colorimetric fliers for environmental monitoring [115]	PLGA, cellulose, environmentally benign colorimetric reagents	Fully biodegradable (partially degraded within 84 days, norm ASTM 1998)	-	EC-autonomous	Sensors	Deployed in artificial ecosystems	-
Shape morphing actuators .	Humidity-responsive blooming actuators [116]	Pollen microgels	Fully biodegradable (no test)	Humidity-responsive materials	EC-autonomous	Actuators	-	-
	Humidity-responsive actuators [117]	Sodium alginate, carbon powder, PVA	Fully biodegradable (no test)	Humidity-responsive materials	EC-autonomous	Actuators	-	-
	Actuators driven by temperature, light, and humidity [118]	CINPs, CNFs, PLA	Fully biodegradable (almost fully degraded after 50 days, test in protease K solution)	Multi-stimuli responsive materials	EC-remotely controlled	Actuators	-	-
	Humidity-responsive self-lifting robots [119]	PCL, PEO, CNC	Fully biodegradable (no test)	Humidity-responsive materials	EC-autonomous	Actuators	-	-

Table S7: Systematic summary of previously published technologies working towards the realization of ESRs highlighting various terrestrial and aquatic environment centered robots and materials.

**Technologies**	**Technology examples**	**Biodegradable material loop**		**Robotic design loop**			**Ecoresorbable robotic loop**	
		Primary materials	Biodegradability	Actuators	Controllability	Components	In-situ robotic test	In-situ eco-resorbable test
Self-burying robots	Humidity-responsive self-burying robots [120]	Wood veneer	Fully biodegradable (no test)	Humidity-responsive materials	EC-autonomous	Actuators	Deployed in the raised bed	-
	Humidity-responsive bio-hybrid actuators for self-burying [121]	Flour, natural awns	Fully biodegradable (no test)	Humidity-responsive materials	EC-autonomous	Actuators	-	-
Pneumatic actuators	Biodegradable suction based grippers [122]	Gelatin hydrogel, cellulose	Fully biodegradable (60% degraded within 10 days, test in wastewater)	Pneumatic actuators	DC-tethered	Actuators	-	-
	Omni-directional and exteroceptive soft manipulators [123]	Gelatin hydrogel, cellulose fiber	Fully biodegradable (almost fully degraded within 14 days, BOD analysis)	Pneumatic actuators	DC-tethered	Actuators, sensors	-	-
	Joint-like vacuum actuators [124]	Egg albumen, saccharides, PLA	Fully biodegradable (no test)	Pneumatic actuators	DC-tethered	Actuators	-	-
	Soft grippers with self-germination capability [125]	Cellulose foam, biodegradable thin-film	Fully biodegradable (no test)	Pneumatic actuators	DC-tethered	Actuators	-	-
Motor driven actuators	Motor-controlled omnidirectional soft manipulators [126]	Gelatin hydrogel with NaCl, cellulose films	Fully biodegradable (98.8% degraded within 56 days, test in soil)	Motors	DC-tethered	Actuators, sensors	-	-
Electrically driven actuators	Biodegradable electro-hydraulic grippers [127]	PLA,vegetable-based oils, gelatin hydrogel with NaCl	Fully biodegradable (almost fully degraded after 50 days, test in compost soil)	Electro-hydraulic actuators	DC-tethered	Actuators	-	-
	Biodegradable electro-hydraulic lifters [128]	PLA, PBAT, soybean oil, gelatin hydrogel with NaCl	Fully biodegradable (partially degraded after 30 days, test in artificial industrial compost soil)	Electro-hydraulic actuators	DC-tethered	Actuators	-	-
Aquatic robots	Aquatic hydraulic hydrogel grippers [129]	Alginate hydrogel	Fully biodegradable (fully degraded within 7 days, test in artificial marine reef aquarium)	Hydraulic actuators	Hybrid EC-DC-tethered	Actuators	-	-
	Aquatic hydraulic water-resistant grippers [130]	KGM	Fully biodegradable (fully degraded within 14 days, test in seawater)	Hydraulic actuators	DC-tethered	Actuators	-	-
	Aquatic swimming robots driven by chemical reaction [131]	Fish feed pellets, gelatin, citric acid, sodium bicarbonate, propylene glycol	Fully biodegradable (no test)	Pneumatic actuators driven by chemical reaction	EC-autonomous	Actuators, energy supply	-	-
